# Supplementary material for: Genetic variation in chromatin state across multiple tissues in Drosophila melanogaster
Source: PLoS Genet. 2023 May 5;19(5):e1010439. doi: 10.1371/journal.pgen.1010439 (PMC10191298; doi:10.1371/journal.pgen.1010439)
Supplement: S1 Table — All eight strains are P-element and Wolbachia free, were brother sister mated for up to 18 generations, and are highly isogenic [48]. Each strain, bar B7, is associated with a reference quality de novo genome assembly [43]. The Stock Number is the Bloomington (‘b’) or Tucson/San Diego (‘t’) Drosophila Stock Center code, although these strains are no longer available from these centers. The stock Full Name, if any, is also given. (DOCX) [file pgen.1010439.s001.docx]

*Supplementary Table 1*

| **Name** | **Stock Number** | **Full Name** | **Collection details** |
| --- | --- | --- | --- |
| A4 | b.3852 | KSA 2 | Koriba Dam, Zimbabwe, 1963 |
| A5 | b.3875 | VAG 1 | Athens, Greece, 1965 |
| A6 | b.3886 | Wild 5B | Red Top Mountain, Georgia, USA, 1966 |
| A7 | t.14021-0231.7 | - | Ken-ting,Taiwan, 1968 |
| B2 | b. 3846 | CA 1 | Cape town, South Africa, 1954 |
| B3 | b.3864 | QI 2 | Israel, 1954 |
| B6 | t.14021-0231.1 | - | Ica, Peru, 1956 |
| B7 | t.14021-0231.4 | - | Kuala Lumpur, Malaysia, 1962 |
