# Supplementary material for: Genetic variation in chromatin state across multiple tissues in Drosophila melanogaster
Source: PLoS Genet. 2023 May 5;19(5):e1010439. doi: 10.1371/journal.pgen.1010439 (PMC10191298; doi:10.1371/journal.pgen.1010439)
Supplement: S2 Table — (DOCX) [file pgen.1010439.s002.docx]

*Supplementary Table 2:*

| **Chromosome** | **Euchromatin Start** | **Euchromatin End** |
| --- | --- | --- |
| 2L | 82455 | 22011009 |
| 2R | 5398184 | 24684540 |
| 3L | 158639 | 22962476 |
| 3R | 4552934 | 31845060 |
| X | 277911 | 22628490 |
