# Supplementary material for: Genetic variation in chromatin state across multiple tissues in Drosophila melanogaster
Source: PLoS Genet. 2023 May 5;19(5):e1010439. doi: 10.1371/journal.pgen.1010439 (PMC10191298; doi:10.1371/journal.pgen.1010439)
Supplement: S3 Table — The Genome column is the percent of each feature type in the genome. (DOCX) [file pgen.1010439.s003.docx]

*Supplementary Table 3:*

| **Feature** | **Brain** | **Ovary** | **Eye Disc** | **Wing Disc** | **Genome** |
| --- | --- | --- | --- | --- | --- |
| Total count | 25464 | 18111 | 18496 | 17413 | na |
| TSS | 27.9% | 39% | 37.5% | 38.2% | 16.2% |
| TTS | 8.6% | 10.5% | 10.3% | 10.2% | 11.3% |
| Exon | 2.9% | 3.7% | 3.1% | 3.3% | 12.9% |
| 5' UTR | 1.6% | 1.7% | 2.1% | 2.2% | 1.3% |
| 3' UTR | 0.8% | 1.0% | 1.0% | 1.0% | 2.0% |
| Intron | 39.5% | 30.9% | 30.5% | 30.0% | 36.7% |
| Intergenic | 17.7% | 12.3% | 14.4% | 14.1% | 18.8% |
| non-coding RNA | 1.0% | 0.8% | 1.1% | 1.1% | 0.9% |
