# Supplementary material for: Genetic variation in chromatin state across multiple tissues in Drosophila melanogaster
Source: PLoS Genet. 2023 May 5;19(5):e1010439. doi: 10.1371/journal.pgen.1010439 (PMC10191298; doi:10.1371/journal.pgen.1010439)
Supplement: S4 Table — (DOCX) [file pgen.1010439.s004.docx]

*Supplementary Table 4:*

| **Geno-type** | **tissue** | **rep** | **N map read pairs** | **N Q30 mapped** | **Pass QC** | **N euchrom Q30** | **N euchrom Q30 SV correct** |
| --- | --- | --- | --- | --- | --- | --- | --- |
| A4 | BR | 1 | 71771158 | 52274422 | pass | 44045024 | 39906836 |
| A4 | BR | 2 | 62368290 | 48936438 | pass | 39888632 | 35951940 |
| A4 | BR | 3 | 75783419 | 53540250 | pass | 46957102 | 42648788 |
| A5 | BR | 1 | 93960376 | 66682740 | pass | 60104462 | 54641732 |
| A5 | BR | 2 | 104946756 | 78893144 | pass | 69588914 | 63294036 |
| A5 | BR | 3 | 74716529 | 61709316 | pass | 54206298 | 49143044 |
| A6 | BR | 1 | 60036748 | 35474110 | pass | 30280784 | 27372406 |
| A6 | BR | 2 | 50750255 | 39838160 | pass | 34505018 | 31120250 |
| A6 | BR | 3 | 69539697 | 47686952 | pass | 41529350 | 37706314 |
| A7 | BR | 1 | 81170471 | 65444566 | pass | 56952234 | 51674972 |
| A7 | BR | 2 | 95112122 | 76447960 | pass | 64960418 | 59009104 |
| A7 | BR | 3 | 81603713 | 56964342 | pass | 51014078 | 46486386 |
| B2 | BR | 1 | 98211903 | 81500160 | pass | 71810664 | 65186390 |
| B2 | BR | 2 | 59244102 | 50617958 | pass | 44335324 | 40220006 |
| B2 | BR | 3 | 61223391 | 52463710 | pass | 45736186 | 41560158 |
| B3 | BR | 1 | 48140599 | 34596332 | pass | 30134474 | 27262448 |
| B3 | BR | 2 | 47302786 | 30924424 | pass | 26149100 | 23611046 |
| B3 | BR | 3 | 58958036 | 36076368 | pass | 31083868 | 28169286 |
| B6 | BR | 1 | 75492051 | 58128380 | pass | 51409164 | 46875760 |
| B6 | BR | 2 | 53692598 | 45076220 | pass | 39648610 | 35912142 |
| B6 | BR | 3 | 66214673 | 43063540 | pass | 36645936 | 33227368 |
| B7 | BR | 1 | 100137834 | 64327562 | pass | 55669962 | 49994944 |
| B7 | BR | 2 | 43326751 | 34532064 | pass | 30298136 | 27062024 |
| B7 | BR | 3 | 40448122 | 31455402 | pass | 27907736 | 25190998 |
| A4 | ED | 1 | 80656000 | 59815788 | pass | 52992504 | 48309038 |
| A4 | ED | 2 | 61685334 | 49750814 | pass | 44107418 | 40239934 |
| A4 | ED | 3 | 91787435 | 73777002 | pass | 64862128 | 59050680 |
| A5 | ED | 1 | 96264676 | 77976884 | pass | 68514152 | 62077086 |
| A5 | ED | 2 | 56723122 | 32797120 | pass | 28439586 | 25709724 |
| A5 | ED | 3 | 22048156 | 18520246 | pass | 16160534 | 14711476 |
| A6 | ED | 1 | 98103076 | 79547840 | pass | 69447044 | 62939648 |
| A6 | ED | 2 | 80190328 | 68546420 | pass | 60569824 | 54744804 |
| A6 | ED | 3 | 112075924 | 92510282 | pass | 81750116 | 73876196 |
| A7 | ED | 1 | 76871348 | 63396382 | pass | 55405028 | 50246964 |
| A7 | ED | 2 | 87796280 | 70520590 | pass | 61553424 | 55931196 |
| A7 | ED | 3 | 99631145 | 70239416 | pass | 61033526 | 55545294 |
| B2 | ED | 1 | 75581524 | 55412894 | pass | 47953644 | 43423182 |
| B2 | ED | 2 | 69879775 | 60423182 | pass | 52980234 | 47985102 |
| B2 | ED | 3 | 67310535 | 57811538 | pass | 50663066 | 45951628 |
| B3 | ED | 1 | 50705365 | 39819642 | pass | 34722654 | 31549212 |
| B3 | ED | 2 | 83872346 | 69014394 | pass | 60078854 | 54296438 |
| B3 | ED | 3 | 70051686 | 58709896 | pass | 51575088 | 46676514 |
| B6 | ED | 1 | 80196358 | 66764068 | pass | 58238558 | 52980476 |
| B6 | ED | 2 | 81189648 | 66477162 | pass | 57788830 | 52440592 |
| B6 | ED | 3 | 69560875 | 56079856 | pass | 49220370 | 44807844 |
| B7 | ED | 1 | 70660653 | 59552736 | pass | 51445154 | 45967382 |
| B7 | ED | 2 | 46142279 | 39290060 | pass | 34717954 | 31347758 |
| B7 | ED | 3 | 76766961 | 59126162 | pass | 51841356 | 46522222 |
| A4 | OV | 1 | 81532698 | 62735178 | pass | 52623390 | 47337012 |
| A4 | OV | 2 | 58997754 | 45029450 | pass | 37189040 | 33327282 |
| A4 | OV | 3 | 103428127 | 81046954 | pass | 68790262 | 61811300 |
| A5 | OV | 1 | 77113758 | 63529554 | pass | 54558530 | 49299252 |
| A5 | OV | 2 | 59234650 | 46582400 | pass | 38048650 | 34327612 |
| A5 | OV | 3 | 76530838 | 55396006 | pass | 44949670 | 40410692 |
| A6 | OV | 1 | 75719086 | 62022436 | pass | 52350264 | 47158736 |
| A6 | OV | 2 | 109355567 | 81718154 | pass | 68233836 | 61612174 |
| A6 | OV | 3 | 89618699 | 68649814 | pass | 57097412 | 51406606 |
| A7 | OV | 1 | 89598938 | 71171406 | pass | 60046320 | 54176252 |
| A7 | OV | 2 | 105624957 | 80165870 | pass | 66653750 | 59823442 |
| A7 | OV | 3 | 91350105 | 76028296 | pass | 63641408 | 57227690 |
| B2 | OV | 1 | 66225646 | 54281112 | pass | 45318216 | 40545374 |
| B2 | OV | 2 | 87852534 | 72597616 | pass | 61582210 | 55398620 |
| B2 | OV | 3 | 110184396 | 89180084 | pass | 75773800 | 68275370 |
| B3 | OV | 1 | 68449030 | 50788994 | pass | 41882250 | 37674094 |
| B3 | OV | 2 | 71617172 | 56847672 | pass | 47878182 | 42987852 |
| B3 | OV | 3 | 71205534 | 56702676 | pass | 48197708 | 43278522 |
| B6 | OV | 1 | 121770614 | 99565362 | pass | 86695036 | 78192806 |
| B6 | OV | 2 | 85845549 | 71108898 | pass | 60337072 | 54305822 |
| B6 | OV | 3 | 91793419 | 69257402 | pass | 57245346 | 51556746 |
| B7 | OV | 1 | 147722618 | 89146168 | pass | 73717892 | 65795130 |
| B7 | OV | 2 | 79761516 | 60599588 | pass | 50038230 | 44409656 |
| B7 | OV | 3 | 42962580 | 33720300 | pass | 27556906 | 24587532 |
| A4 | WD | 1 | 60515151 | 49738892 | pass | 44243336 | 40315938 |
| A4 | WD | 2 | 56078307 | 45685146 | pass | 40602590 | 37064638 |
| A4 | WD | 3 | 51314785 | 32872384 | pass | 28625726 | 26134694 |
| A5 | WD | 1 | 50354595 | 43154900 | pass | 38044562 | 34770630 |
| A5 | WD | 2 | 45469759 | 35994894 | pass | 31439700 | 28660300 |
| A5 | WD | 3 | 65988568 | 52040818 | pass | 45442736 | 41354866 |
| A6 | WD | 1 | 64728252 | 47195092 | pass | 41612180 | 37933684 |
| A6 | WD | 2 | 82221797 | 70455610 | pass | 62819410 | 57102528 |
| A6 | WD | 3 | 93572161 | 78823382 | pass | 69735446 | 63243370 |
| A7 | WD | 1 | 61092104 | 52066686 | pass | 45519698 | 41418234 |
| A7 | WD | 2 | 84757693 | 71119418 | pass | 62719728 | 57210128 |
| A7 | WD | 3 | 86537558 | 70314768 | pass | 61882494 | 56504754 |
| B2 | WD | 1 | 83582025 | 66927970 | pass | 58428114 | 52995220 |
| B2 | WD | 2 | 85742323 | 73722046 | pass | 65226462 | 59299268 |
| B2 | WD | 3 | 52279250 | 45647482 | pass | 39921550 | 36149674 |
| B3 | WD | 1 | 20539457 | 7216490 | fail | NA | NA |
| B3 | WD | 2 | 68864938 | 58342154 | pass | 51721966 | 47097194 |
| B3 | WD | 3 | 53466204 | 44496162 | pass | 38890938 | 35325288 |
| B6 | WD | 1 | 93000699 | 77663020 | pass | 68424846 | 62396890 |
| B6 | WD | 2 | 71531414 | 57303772 | pass | 50990114 | 46560980 |
| B6 | WD | 3 | 59763212 | 50287802 | pass | 44357814 | 40478002 |
| B7 | WD | 1 | 54458465 | 46577628 | pass | 40953970 | 36921480 |
| B7 | WD | 2 | 42220164 | 36215608 | pass | 31970698 | 28937010 |
| B7 | WD | 3 | 57232749 | 49048528 | pass | 43086702 | 38768168 |
