# Supplementary figures and images for: Genetic variation in chromatin state across multiple tissues in Drosophila melanogaster
Source: PLoS Genet. 2023 May 5;19(5):e1010439. doi: 10.1371/journal.pgen.1010439 (PMC10191298; doi:10.1371/journal.pgen.1010439)

*Supplementary Figure 1:*


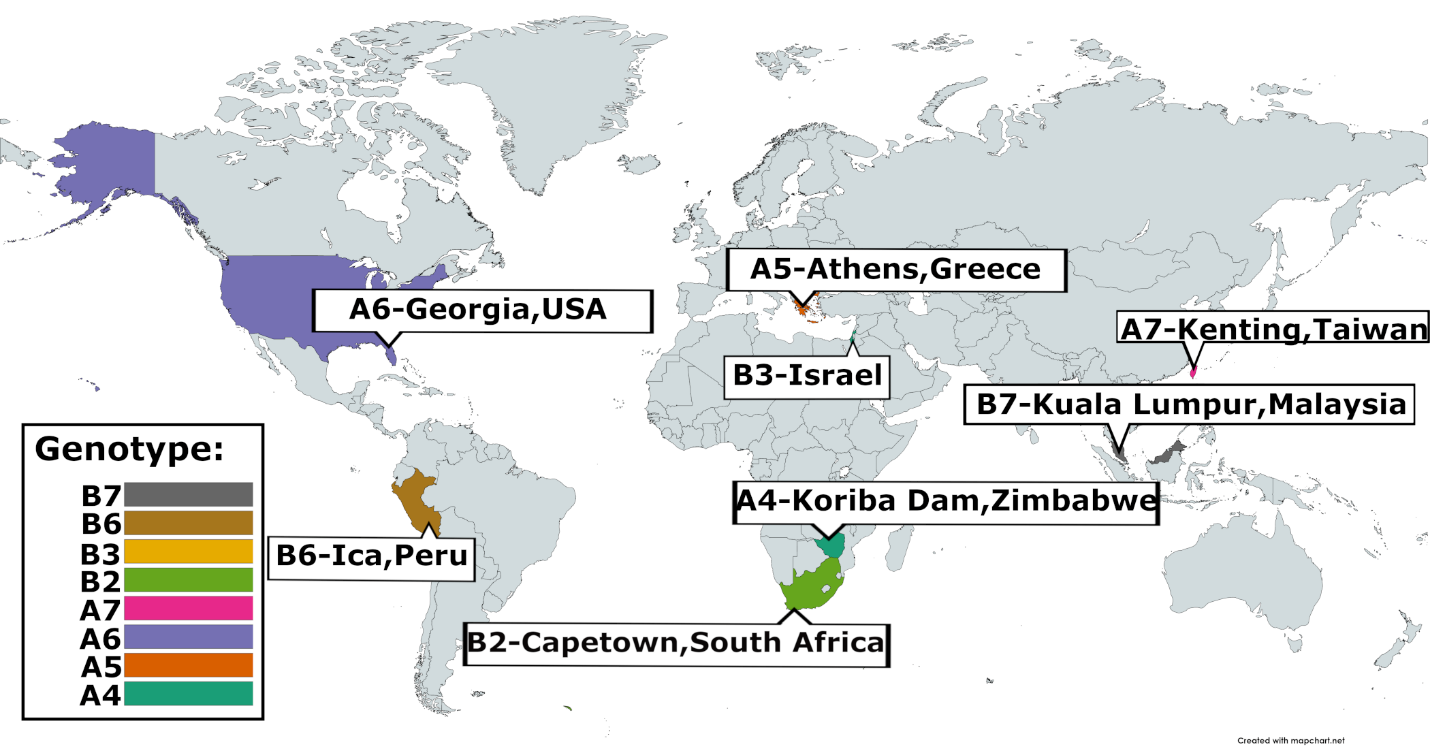

Supplement: S1 Fig — The color legend for genotypes is also kept constant throughout the paper. This map was created using mapchart.net, licensed under This work is licensed under a Creative Commons Attribution-ShareAlike 4.0 International License (CC BY-SA 4.0). (DOCX) [file pgen.1010439.s005.docx]

*Supplementary Figure 2:*


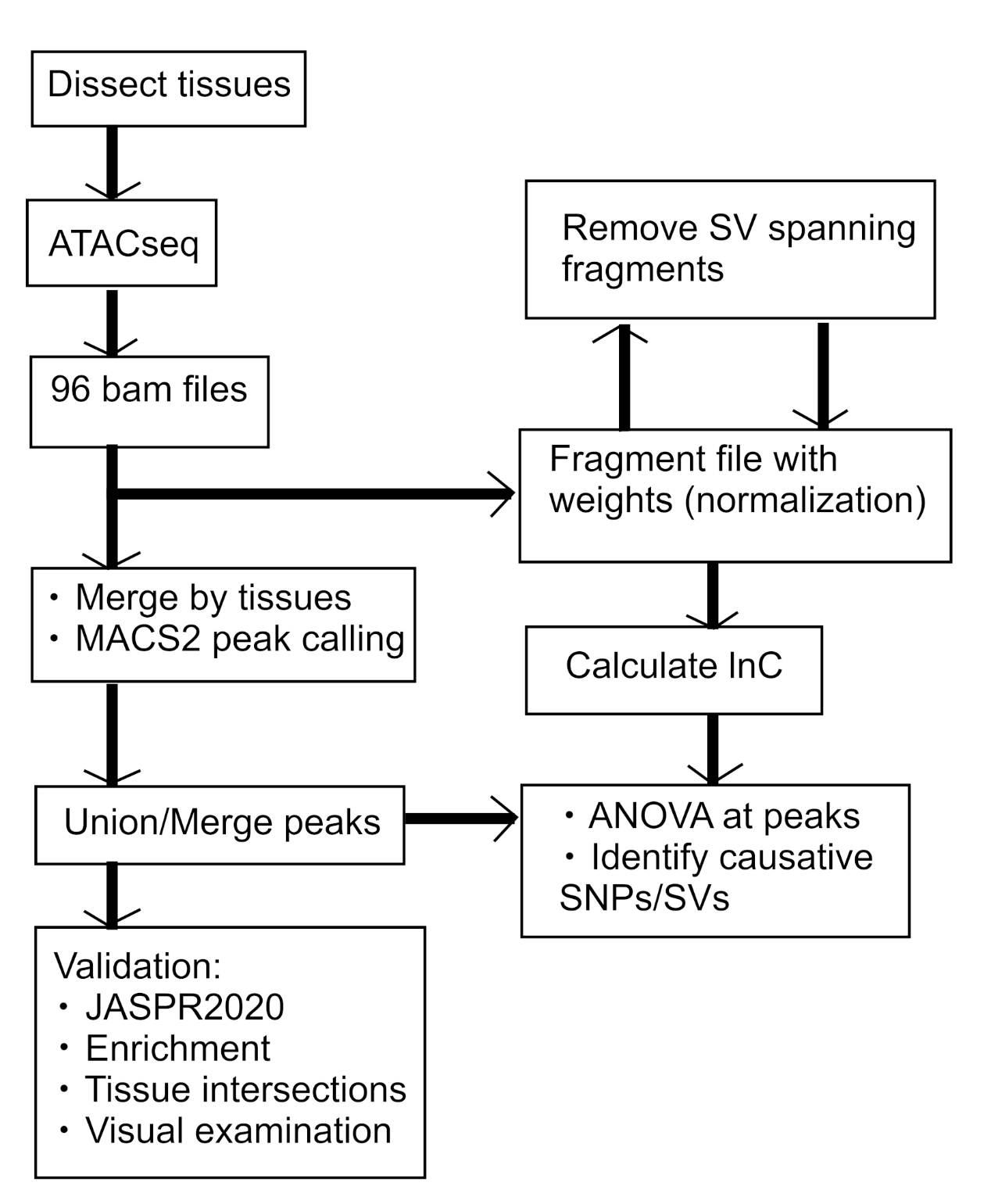

Supplement: S2 Fig — (DOCX) [file pgen.1010439.s006.docx]

*Supplementary Figure 3:*


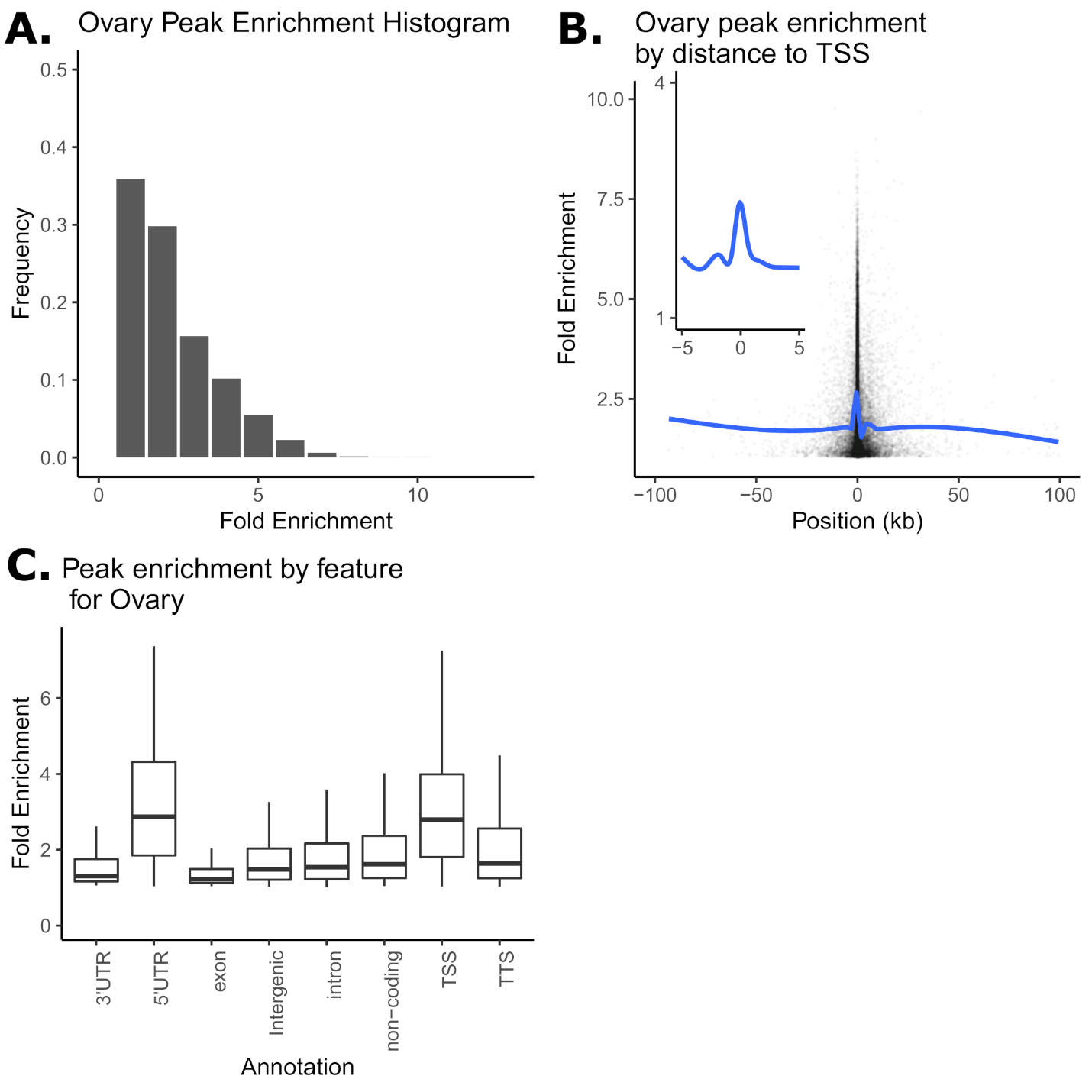

Supplement: S3 Fig — (A) Distribution of peak enrichment scores for the ovary samples. (B): Peak enrichment scores as a function of distance to the nearest transcription start site with a smoothing line for the ovary samples. Insert focuses on peaks within 10kb of the TSS and showing only the smoothing line. (C): Peak enrichment distribution as a function of genomic feature for the ovary samples. (DOCX) [file pgen.1010439.s007.docx]

*Supplementary Figure 4:*


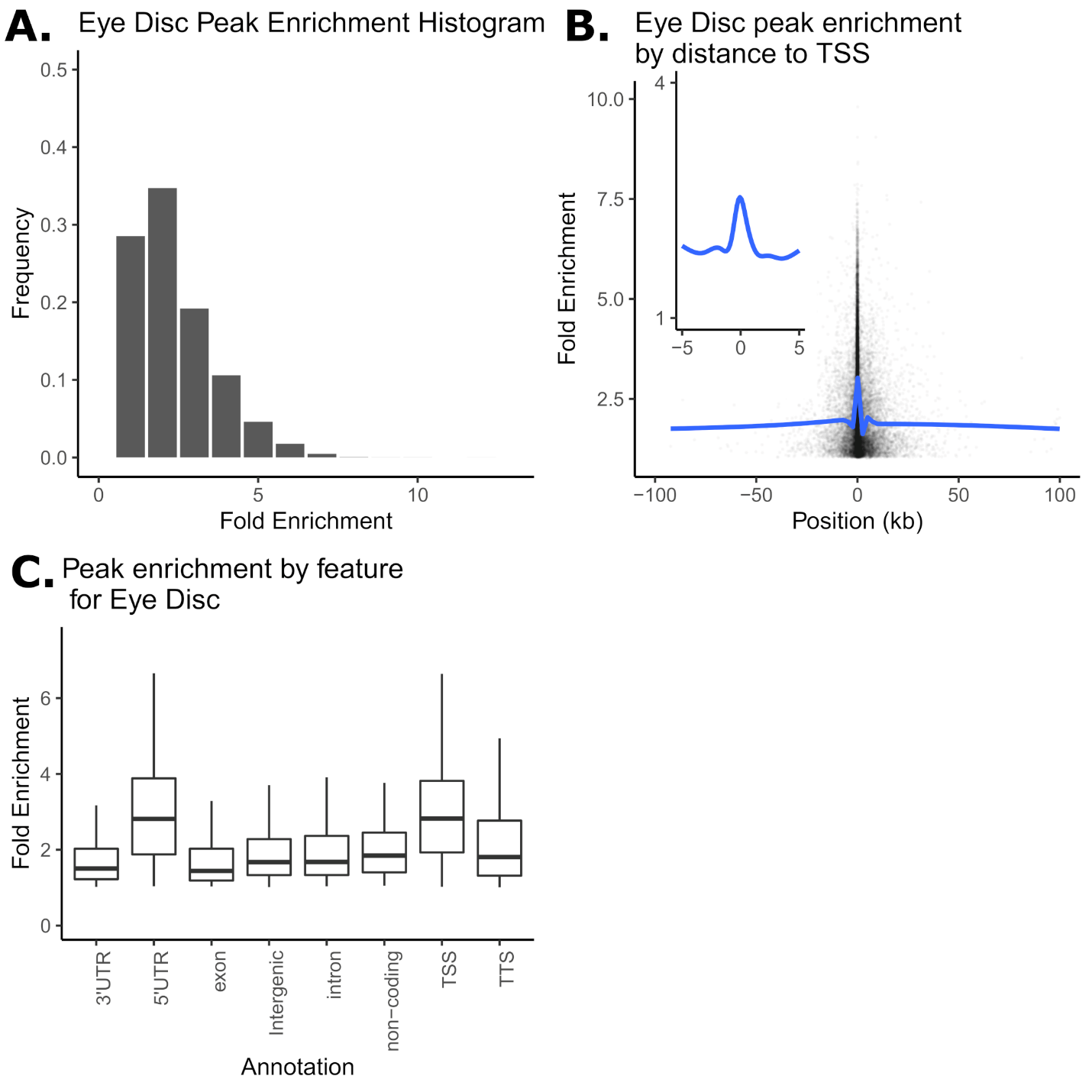

Supplement: S4 Fig — (A) Distribution of peak enrichment scores for the eye disc samples. (B): Peak enrichment scores as a function of distance to the nearest transcription start site with a smoothing line for the eye disc samples. Insert focuses on peaks within 10kb of the TSS and showing only the smoothing line. (C): Peak enrichment distribution as a function of genomic feature for the ovary samples for the eye disc samples. (DOCX) [file pgen.1010439.s008.docx]

*Supplementary Figure 5:*


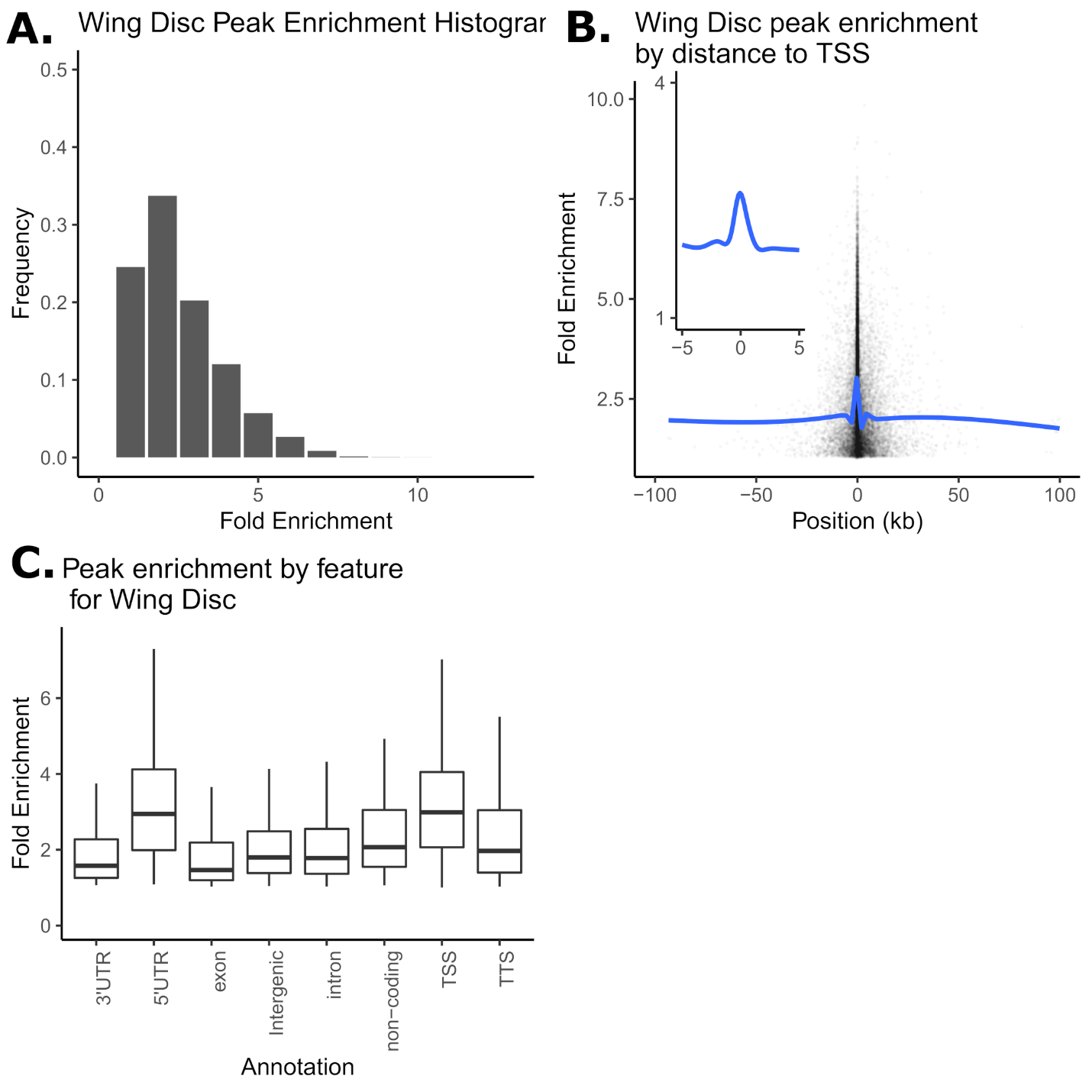

Supplement: S5 Fig — (A) Distribution of peak enrichment scores for the wing disc samples. (B): Peak enrichment scores as a function of distance to the nearest transcription start site with a smoothing line for the wing disc samples. Insert focuses on peaks within 10kb of the TSS and showing only the smoothing line. (C): Peak enrichment distribution as a function of genomic feature for the wing disc samples. (DOCX) [file pgen.1010439.s009.docx]

*Supplementary Figure 6:*


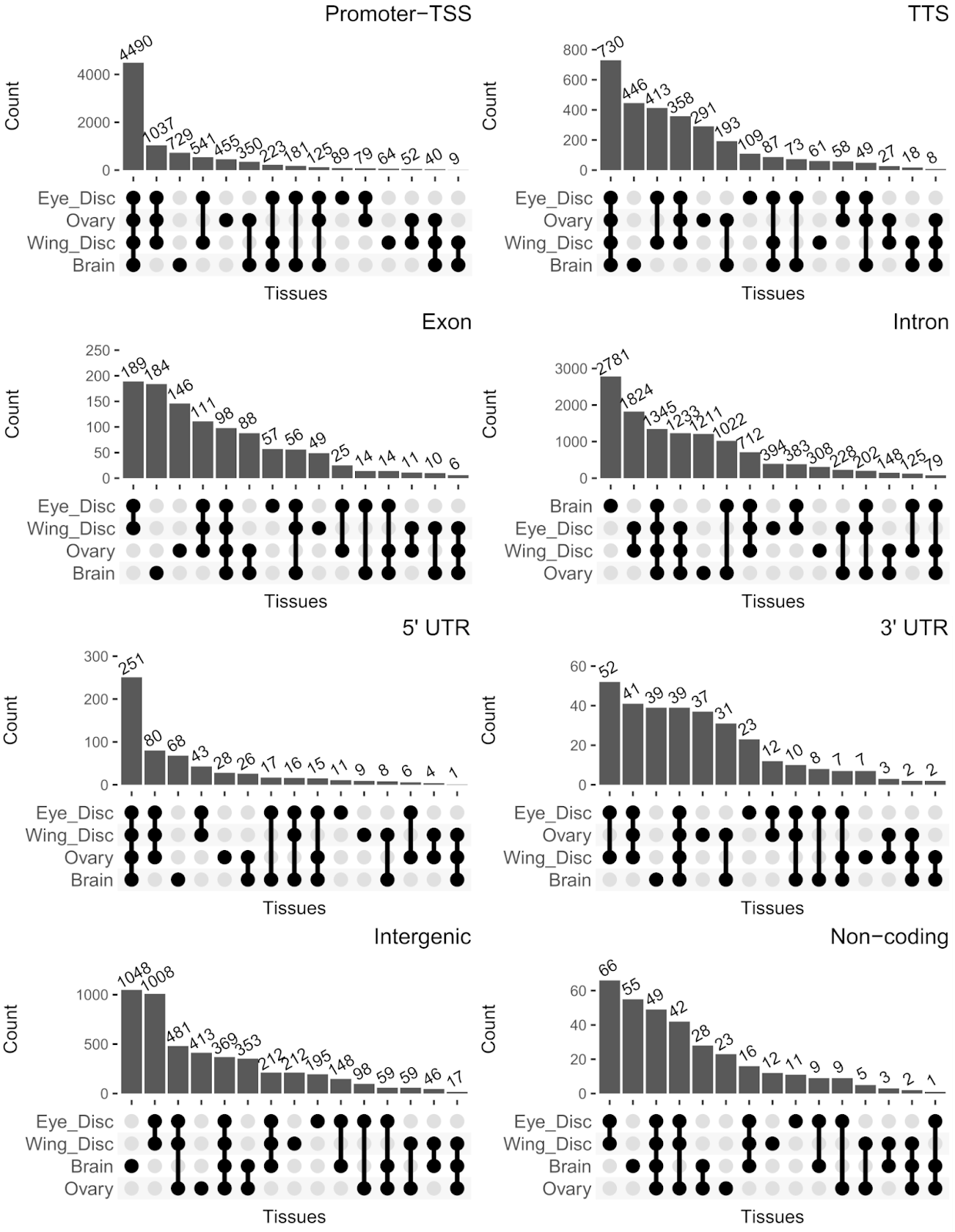

Supplement: S6 Fig — (DOCX) [file pgen.1010439.s010.docx]

*Supplementary Figure 7:*


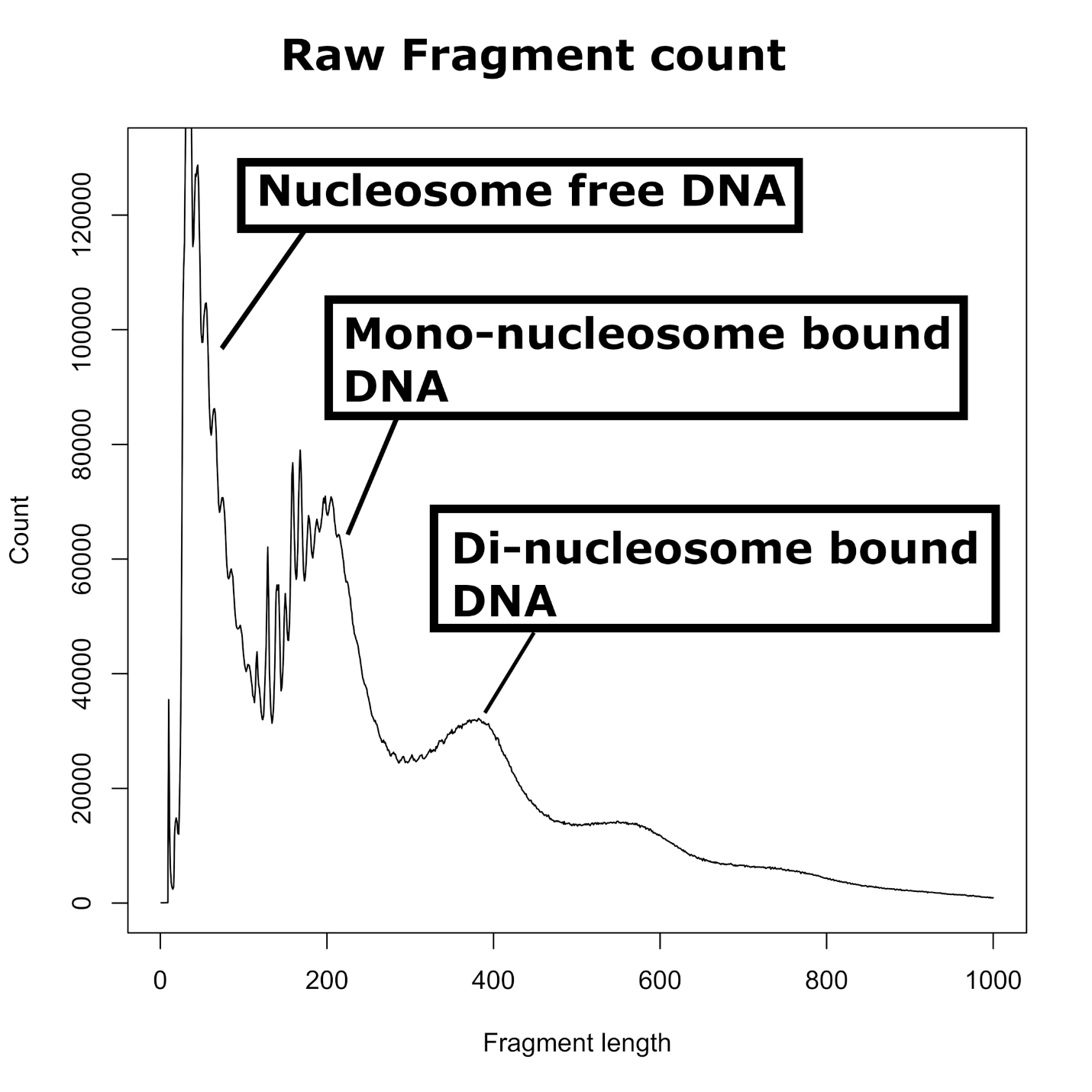

Supplement: S7 Fig — (DOCX) [file pgen.1010439.s011.docx]

*Supplementary Figure 8:*


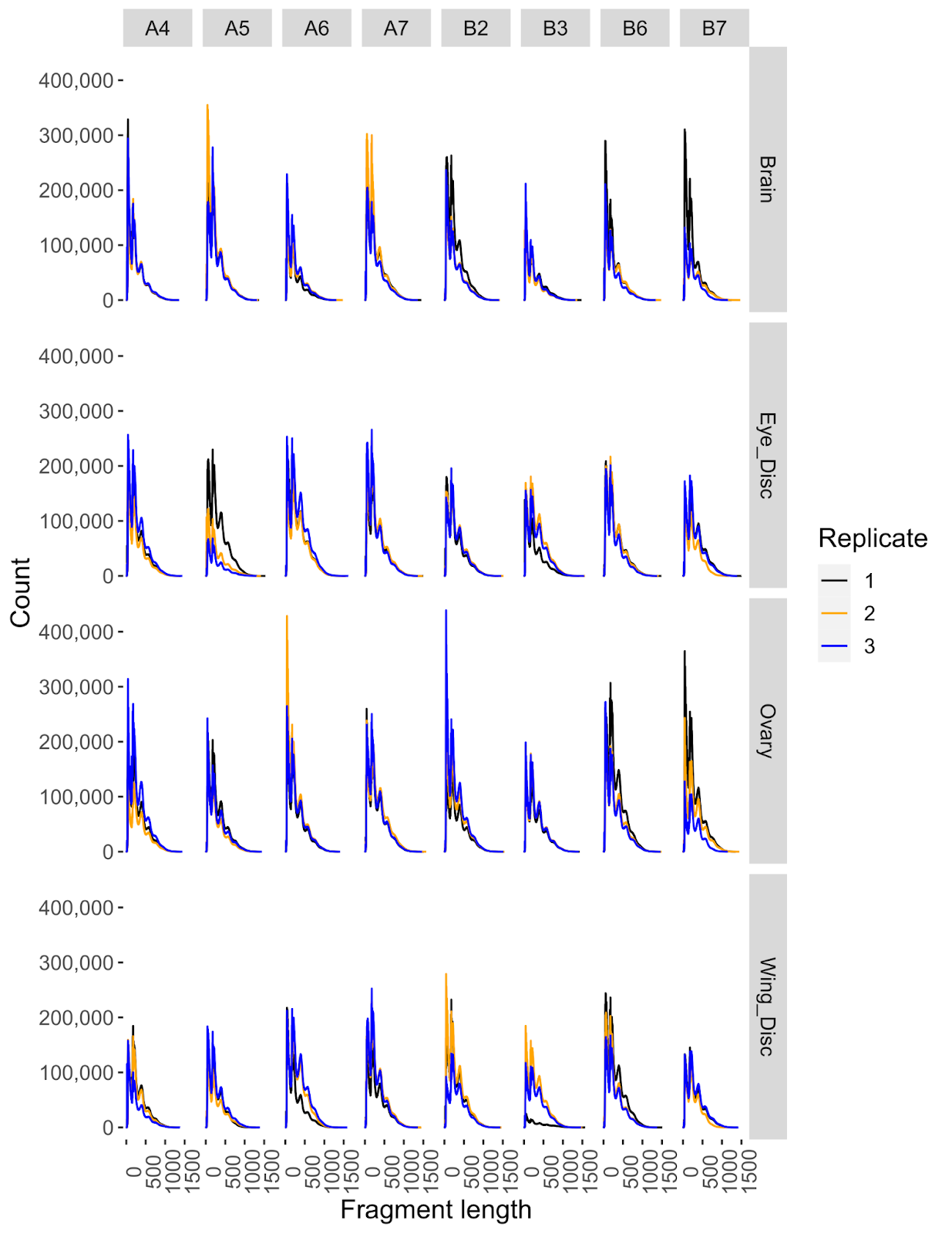

Supplement: S8 Fig — (DOCX) [file pgen.1010439.s012.docx]

*Supplementary Figure 9:*

*
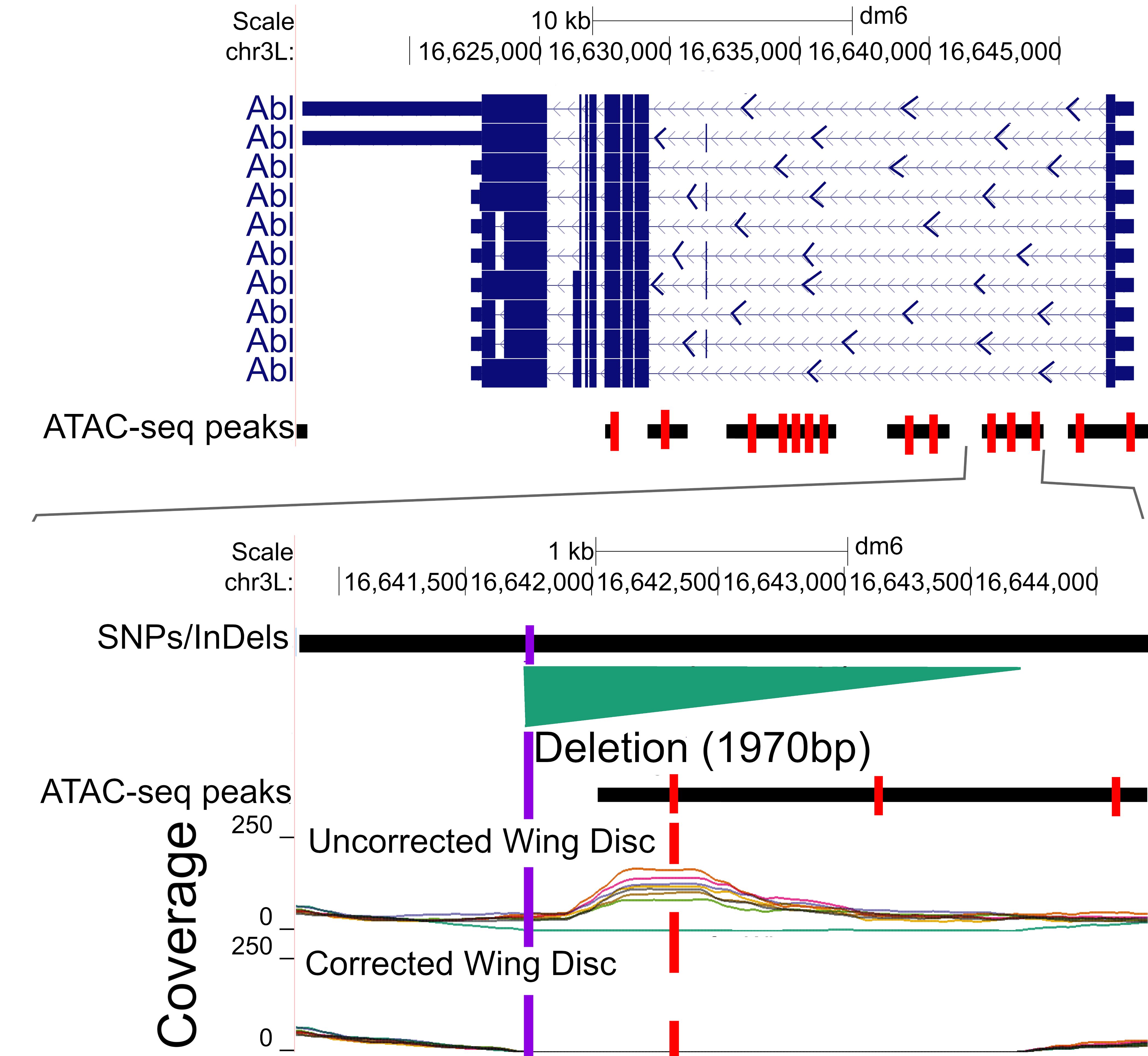
*

Supplement: S9 Fig — (DOCX) [file pgen.1010439.s013.docx]

*Supplementary Figure 10:*


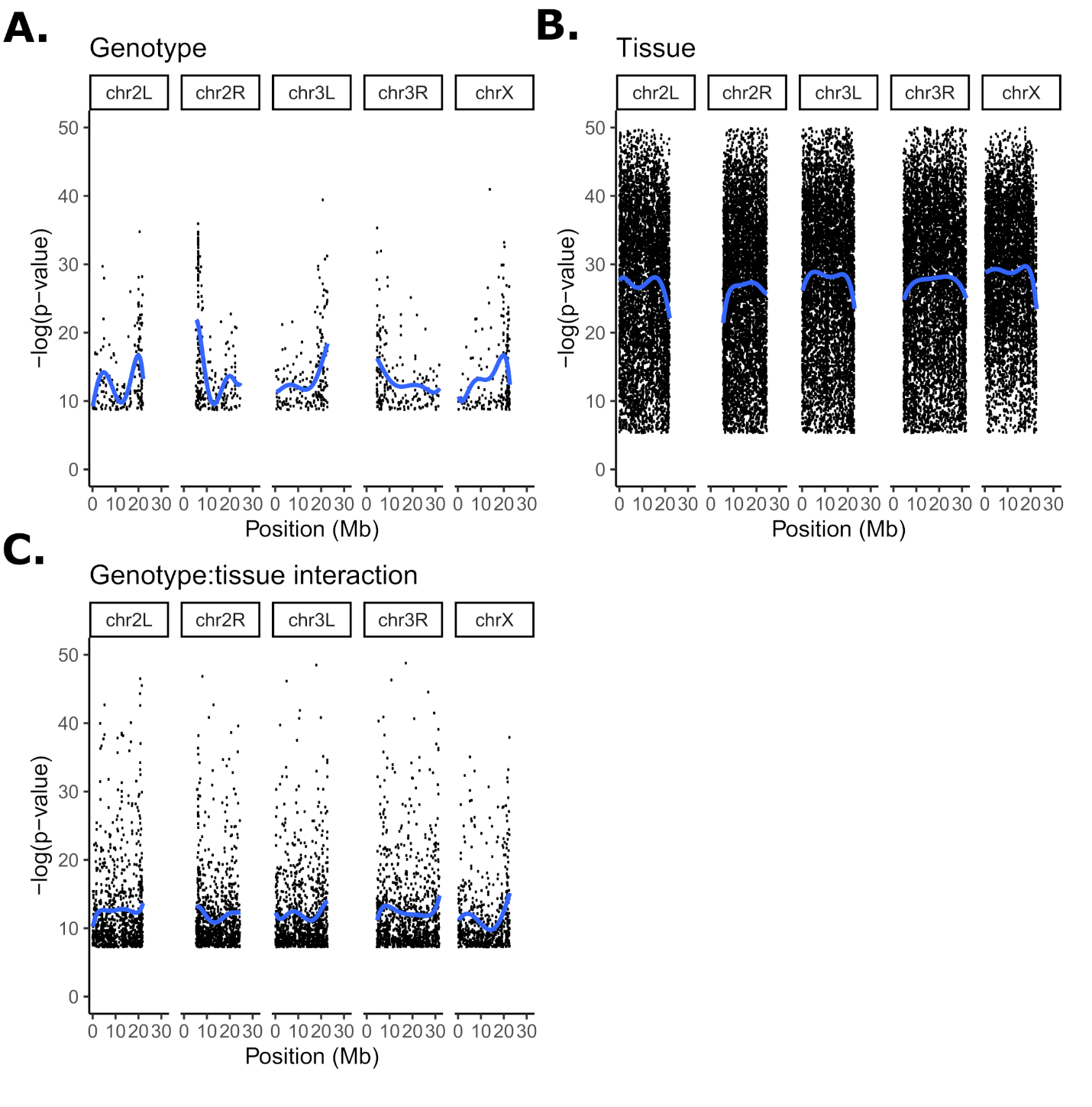

Supplement: S10 Fig — (A): Manhattan plot for significant peaks by genotype. (B): Manhattan plot for significant peaks by tissue. (C): Manhattan plot for significant peaks by genotype and tissue interaction. (DOCX) [file pgen.1010439.s014.docx]

*Supplementary Figure 11:*


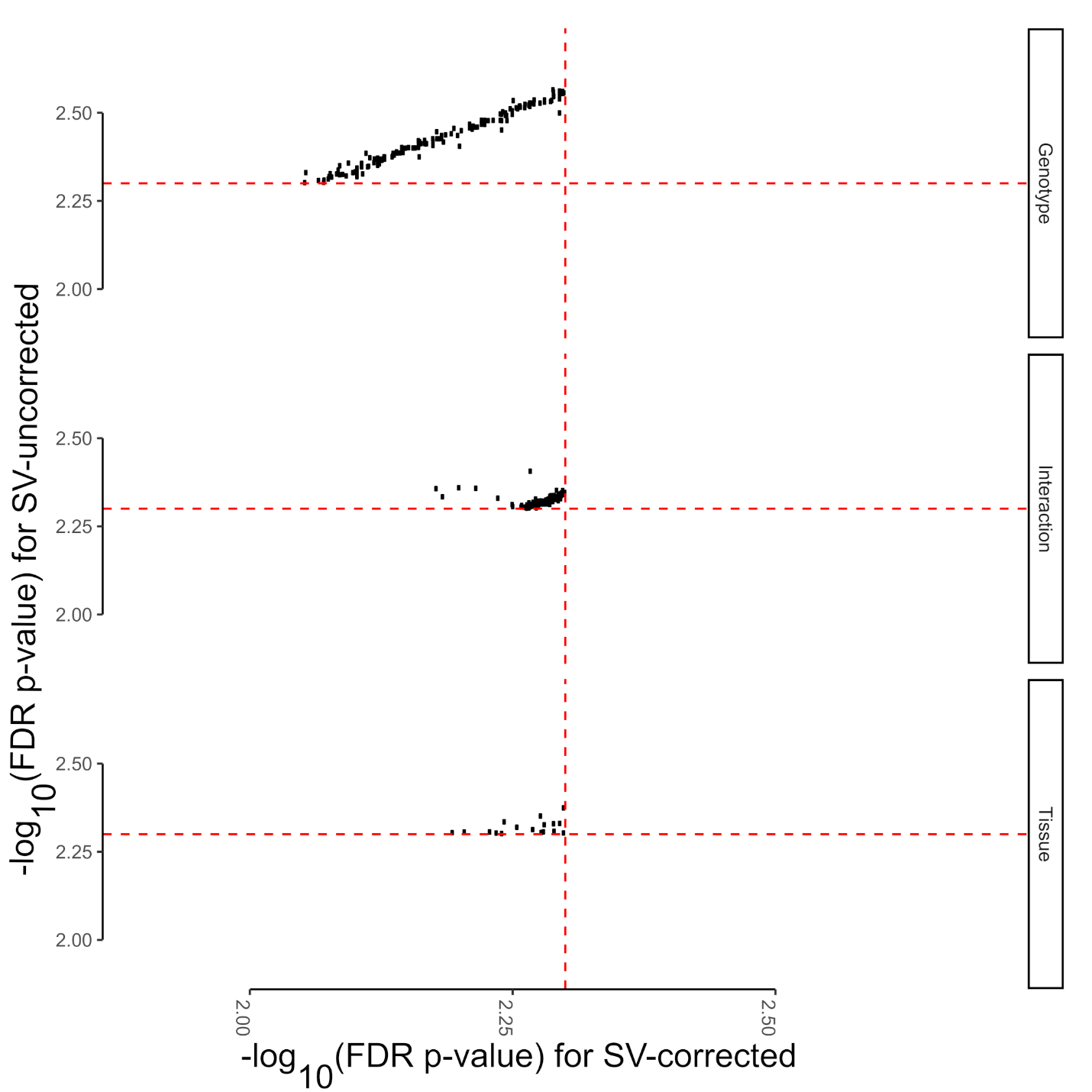

Supplement: S11 Fig — Red dashed lines showing -log(p-value) = 2.3 for SV-uncorrected (horizontal) and SV-corrected (vertical). Sampling variation likely drives the observed differences, as hits tend to be just beyond the significance level in the uncorrected dataset. (DOCX) [file pgen.1010439.s015.docx]

*Supplementary Figure 12:*


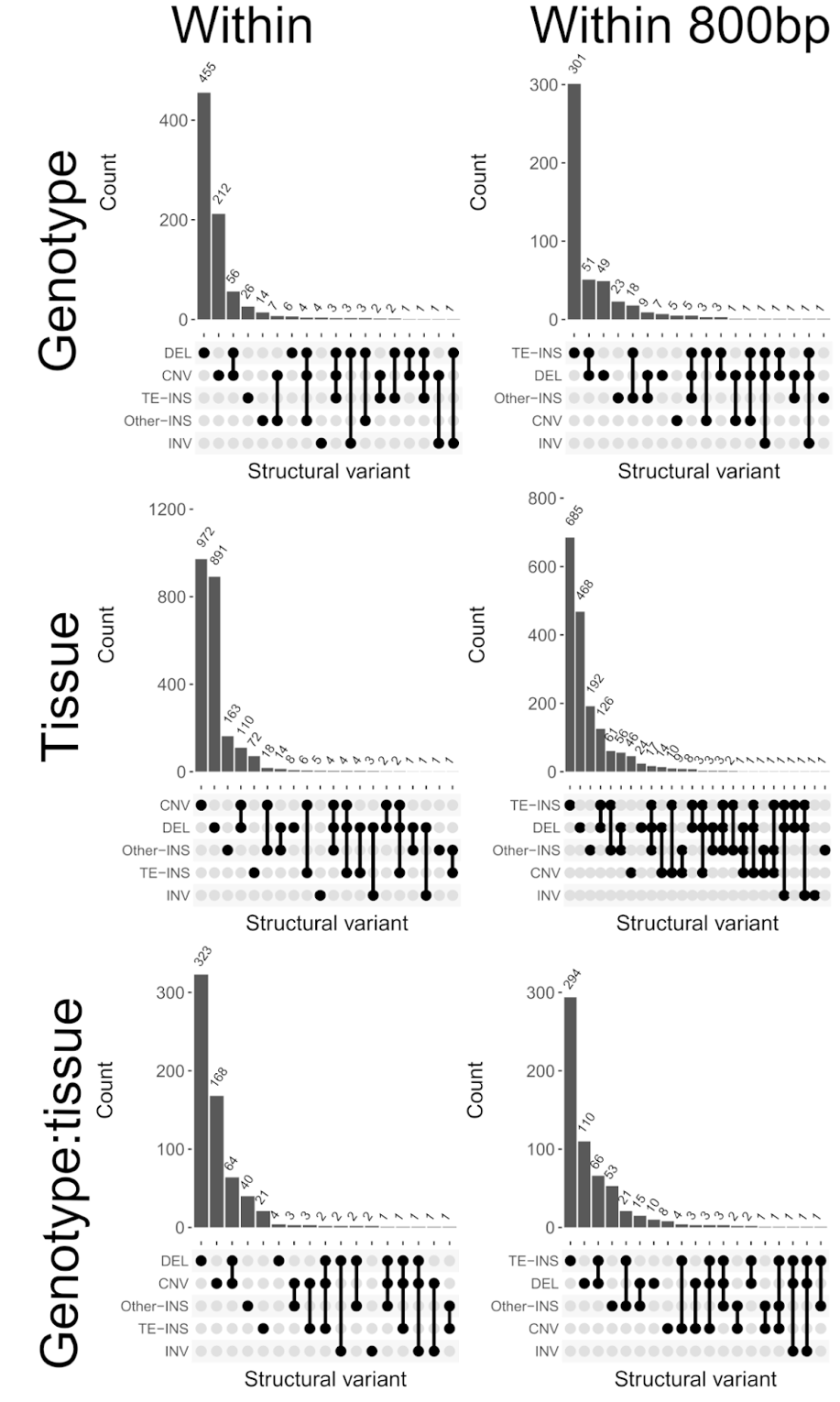


*.*

Supplement: S12 Fig — Variant types are deletion relative to reference (DEL), insertion due to TE (TE-INS), non-TE insertions (Other-INS), inversion (INV), or copy number variant (CNV). The categories are non-exclusive as multiple SV events could be close to an ATAC-seq peak, especial since we integrate over all strains. (DOCX) [file pgen.1010439.s016.docx]

*Supplementary Figure 13:*


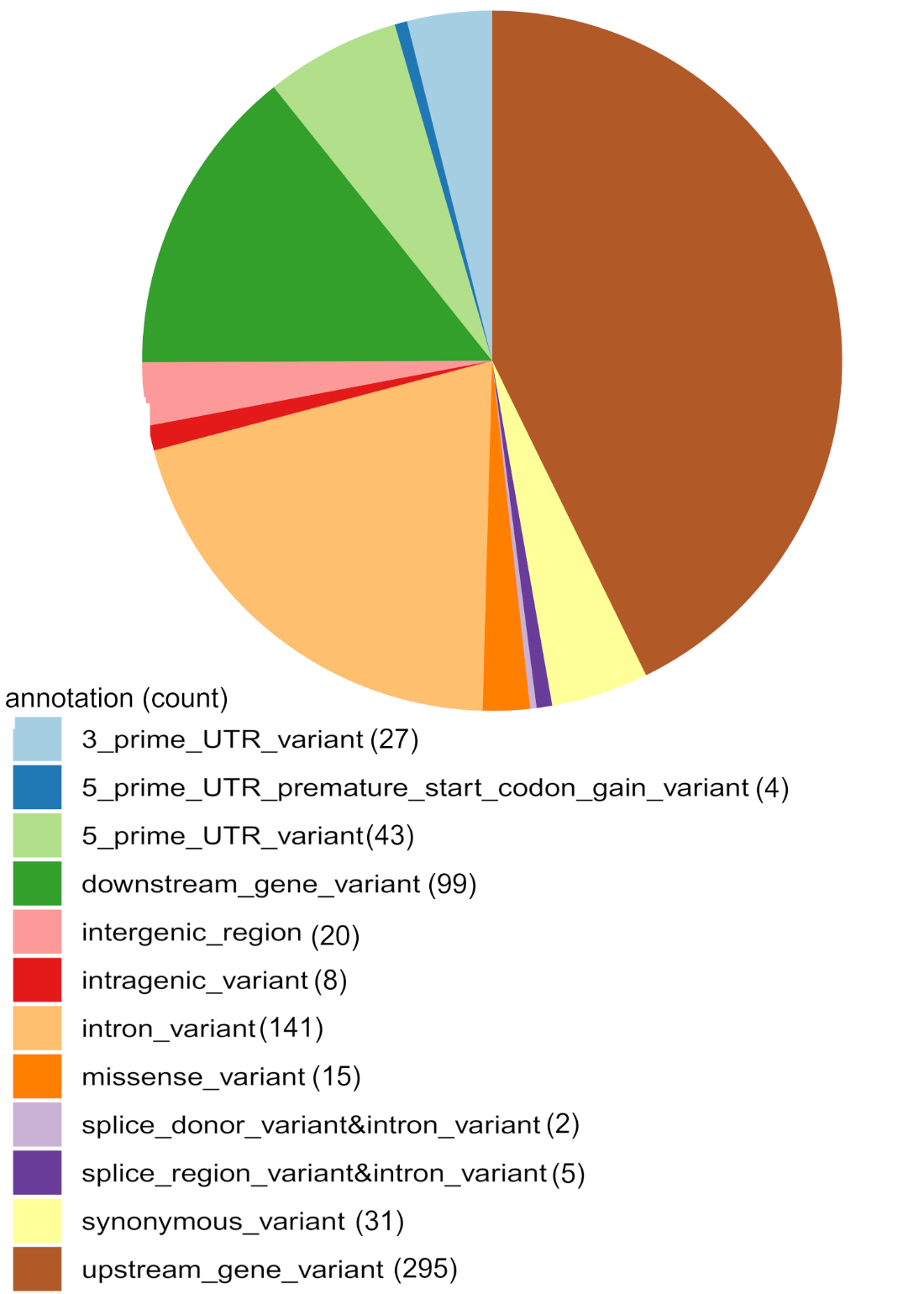

Supplement: S13 Fig — (DOCX) [file pgen.1010439.s017.docx]

*Supplementary Figure 14:*


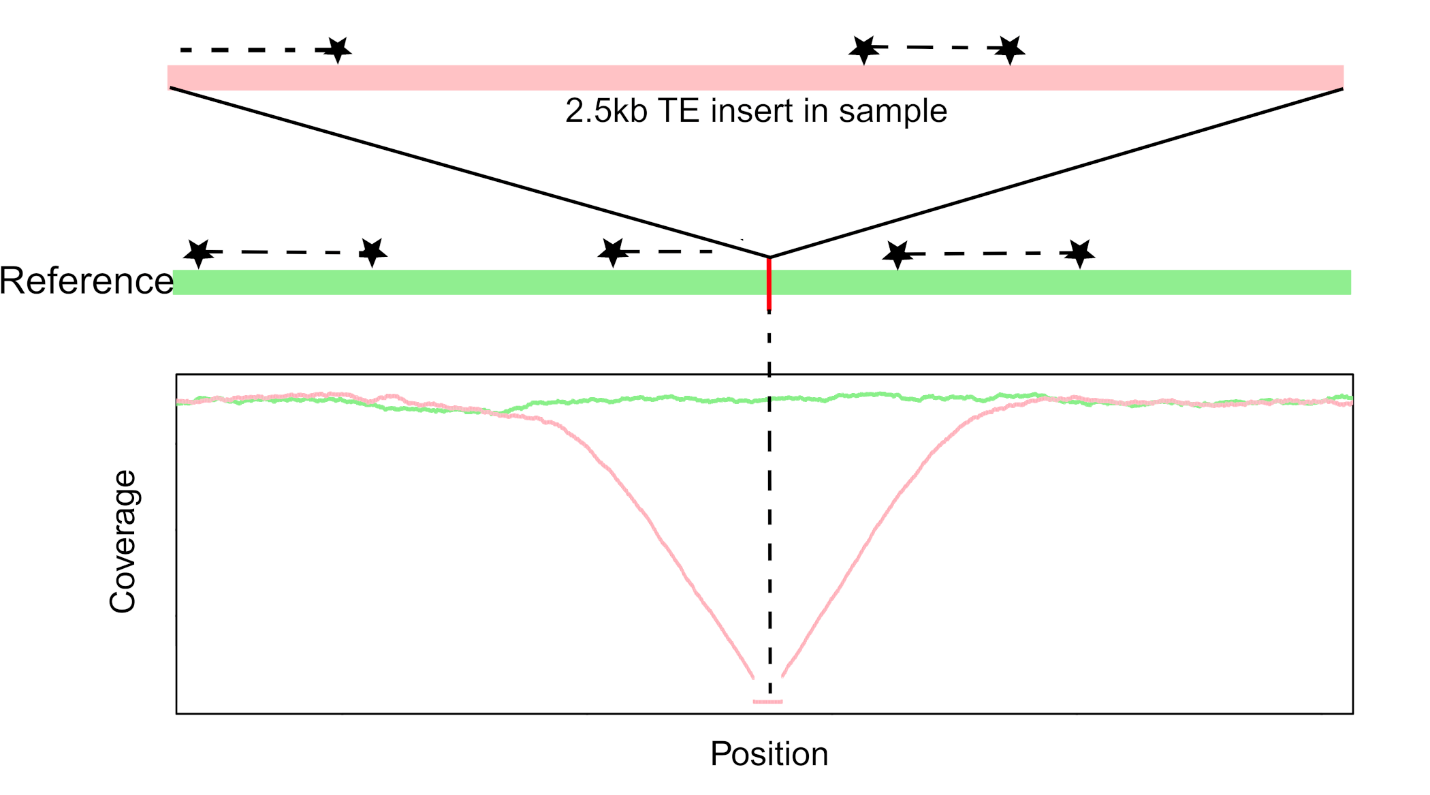

Supplement: S14 Fig — The green track depicts the reference 2kb sequence with the pink track depicting a non-reference 2.5kb transposon insertion (not drawn to scale) at the location of the red dash. The bottom plot depicts the coverage of reference (green) or non-reference sample aligned to the reference genome. The stars depict short read (forward and reverse reads) pairs and dashed lines the fragments created by read pairs. Chimeric fragments with one read in the TE insertion are mis-mapped, resulting in strong dips in read coverage at locations close to the TE insertion site. (DOCX) [file pgen.1010439.s018.docx]
