# Supplementary material for: Genetic variation in chromatin state across multiple tissues in Drosophila melanogaster
Source: PLoS Genet. 2023 May 5;19(5):e1010439. doi: 10.1371/journal.pgen.1010439 (PMC10191298; doi:10.1371/journal.pgen.1010439)

*Supplementary Text 1: ATAC-seq protocol.*

Brittny Smith

Stuart Macdonald

**Nuclei Isolation**

1. Dissect tissue of interest in lysis buffer.

We have successfully started the protocol with:

i. Ovaries from 5 adult, mated females,

ii. Eye-antennal discs from 5 male third-instar larvae,

iii. Wing discs from 5 male third-instar larvae,

iv. Brains from 10 adult, mated females.

Ideally you want to keep everything cold and/or rapidly dissect your tissue out.

The lysis buffer is:

10 mM Tris-HCl, pH 7.4

10 mM NaCl

3 mM MgCl_2_

0.1% IGEPAL CA-630

2. Place into 200-μl lysis buffer on ice in 1.7-ml tube.

3. Manually grind 25 times using a blue plastic pestle (Fisher, K749521-1500).

A "grind" in this case being loosely defined as a single turn of the pestle with your fingers.

4. Let sample sit on ice for ~1-min.

5. Repeat steps 3 and 4 twice each (a total of 75 grinds).

6. Spin sample at 100-g for 10-min at 4ºC.

7. Remove supernatant. Resuspend sample in 200-μl lysis buffer.

8. Run sample through 30-μm filter cloth.

The cloth is Nitex Nylon Mesh (30μm) from Genesee Scientific (Cat # 57-105). After it arrives you will want to wash it in water, rinse with ethanol, dry, and cut into ~1-inch squares.

You'll start this step with a fresh 1.7ml tube in a rack. Remove the pointy end from a standard 1000-μl tip so its got a nice wide bore, and drop into the destination tube. Then on top of the barrel of the tip you can lay a piece of the cloth. Take up your sample from step #7 in a pipette, jam the tip into the cloth (making a little sieve with the barrel of the tip that's in the destination tube) and fire the resuspended cells through the mesh. In step #9 below you're simply ensuring everything went through by adding more lysis buffer to the "sieve".

9. Wash through cloth with 200-μl lysis buffer.

10. Spin sample at 1000-g for 10-min at 4ºC.

11. Pipette off supernatant.

**Tagmentation Reaction**

1. To the cell pellet add 25-μl of tagmentation reaction mix:

12.5μl 2X TD Buffer

1.25μl Tn5 Transposase

11.25μl H_2_O

The TD Buffer and Tn5 Transposase are from the Illumina Nextera DNA Sample Preparation Kit (Cat # FC-121-1030). Now (mid-2021) you can purchase them separately from Illumina.

2. Pipette to resuspend pellet in the tagmentation mix.

3. Incubate for 30-min at 37ºC.

4. Place sample on ice (if moving forward to purification) or freeze at −20ºC.

**Qiagen MinElute Purification**

1. Add 125-μl (5 volumes) of PB buffer to the 25-μl (1 volume) tagmentation reaction, and mix.

Use Qiagen MinElute PCR Purification Kit (Cat # 28004). Ensure that the correct volume of 100% ethanol is added to buffer PE concentrate before use.

2. Place MinElute column in a 2-ml collection tube.

3. Add sample to column, and centrifuge at 18,000-g for 1-min at RT.

4. Discard flow-through and place column back in collection tube.

5. Add 750-μl PE buffer to column, and centrifuge at 18,000-g for 1-min at RT.

6. Discard flow-through and place column back in collection tube.

7. Centrifuge at 18,000-g for 1-min at RT.

8. Place column in new 1.7-ml tube.

9. Add 20-μl EB buffer to the center of the column.

10. Let column stand for 1-min, and centrifuge at 18,000-g for 1-min at RT.

11. Place sample on ice (if moving forward to PCR) or freeze at −20ºC.

**PCR Amplification**

*Reaction*

1 × 25μl reaction

5-μl Purified, tagmented DNA

2.5-μl H_2_O

2.5-μl SJM 7## Indexed primer (@ 12.5-μM)

2.5-μl SJM 5## Indexed primer (@ 12.5-μM)

12.5-μl Kapa Master Mix

For ovary samples we used 5-μl of purified tagmented DNA. For discs and brain samples we used 7.5-μl of purified tagmented DNA and eliminated the water from the reaction.

The Kapa Master Mix comes from Cat # KK2612.

The "SJM 7## Indexed primer" (and the 5## version) are custom versions of Illumina Nextera index primers. (You can obviously use those described in the original ATACseq protocol.)

SJM 7## Indexed primer:

xxxxxxx = 7-base i7 index

5'- CAAGCAGAAGACGGCATACGAGATxxxxxxxGTCTCGTGGGCTCGG -3'

SJM 5## Indexed primer:

yyyyyyyy = 8-base i5 index

5'- AATGATACGGCGACCACCGAGATCTACACyyyyyyyyTCGTCGGCAGCGTC -3'

*Thermocycling*

72ºC 5-min

98ºC 30-sec

12 cycles of:

98ºC 10-sec

63ºC 30-sec

72ºC 1-min

4ºC hold

**Bead Cleanup**

1. Add 25-μl of beads to sample.

This is a 1X bead cleanup. So provides limited size selection. Beads are Agencourt AMPure XP beads (A63881).

2. Mix and incubate at RT for 5-min.

3. Place sample on magnetic plate for 1-min.

4. Remove and discard supernatant.

5. Wash with 50-μl of 70% ethanol. Repeat the wash.

6. Air dry for 5-min.

7. Resuspend in 20-μl of Qiagen EB buffer.

8. Incubate at RT for 2-min.

9. Place sample on magnetic plate for 1-min.

10. Transfer 20-μl to new 1.7-ml tube.

**Quality Control**

1. Qubit sample using BR (broad range) dsDNA kit (ThermoFisher Q32850).

Not totally clear to what extent the Qubit value informs you about sample quality. We have seen nice ATACseq peaks from samples that had <10 ng/μl values, and similar peak profiles from samples that had >40 ng/μl values.

2. Run on Agilent TapeStation instrument using a Genomic DNA ScreenTape.

The TapeStation is similar to the Agilent BioAnalyzer, but a little lower resolution. Example good pictures (meaning that if you see this, and then sequence that library, the sample has peaks and isn't simply an over-digested mess) are below. You want to see the periodicity at the low molecular weight end of the TapeStation profile.


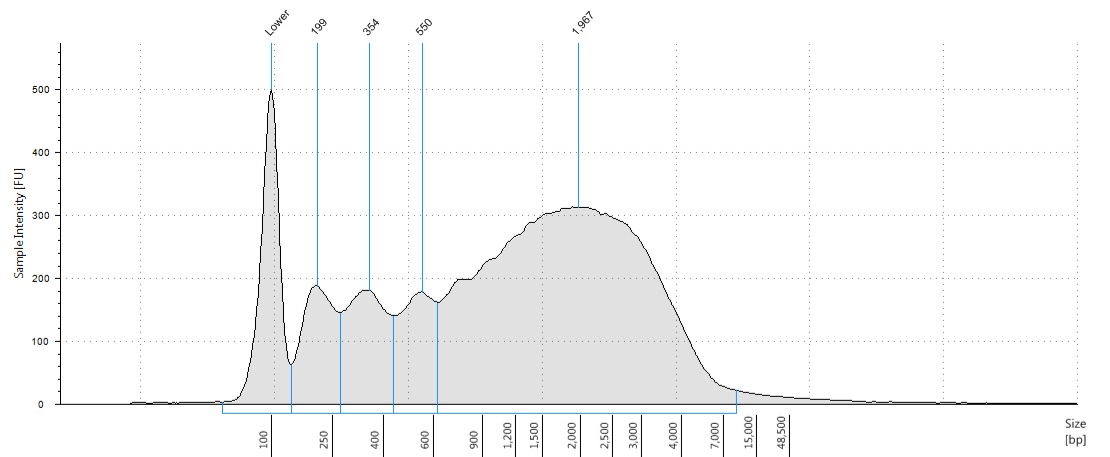


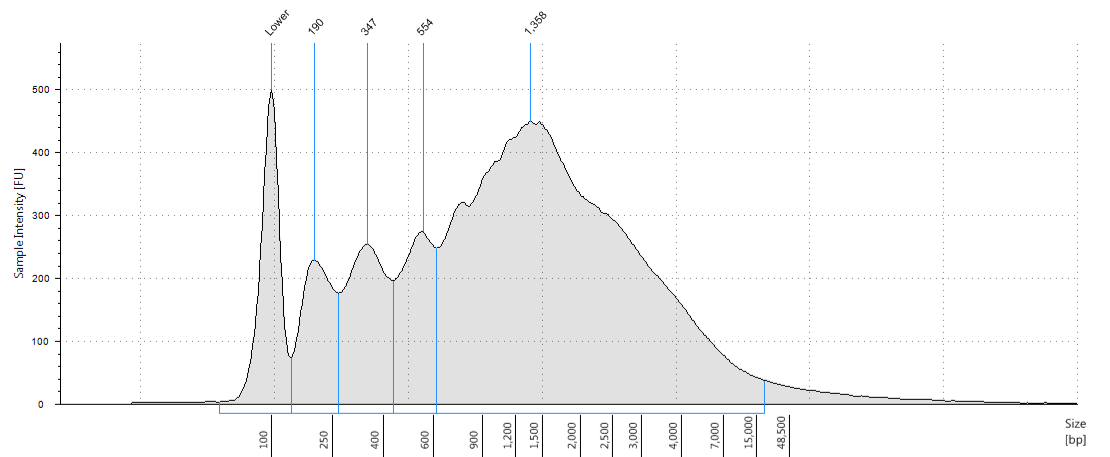

Supplement: S1 Text — (DOCX) [file pgen.1010439.s019.docx]
