# Supplementary material for: Genetic variation in chromatin state across multiple tissues in Drosophila melanogaster
Source: PLoS Genet. 2023 May 5;19(5):e1010439. doi: 10.1371/journal.pgen.1010439 (PMC10191298; doi:10.1371/journal.pgen.1010439)
Supplement: Attachment — Submitted filename: ATACseq_response.pdf [file pgen.1010439.s020.pdf]

Dear Kelly:

Sorry, this took a little longer than I would have liked. Some of the changes required a bit of computational work. And Christmas & New Years occurred. Thank-you for the opportunity to revise the paper.

We have made extensive changes. There are also long comments below explaining the revision a bit better. We have added three supplementary figures (Supp Fig 6,8,&11), and a new supplementary table (Supp Table 4). We have also revised the manuscript itself. We ran the figures through the figure checker software.

What we have done is include a manuscript for your and the reviewers with major changes in BLUE. We have not marked up changes to the supplementary information as the changes consist of new figures and a new table. We have a version with the figures and tables "in-line", thus it resembles the manuscript reviewed fairly closely (which we believe makes it easier to assess if the changes are sufficient). We have a 2nd version with the figures pulled out and no blue formatting (the text is the same).

Tony and Khoi

##################################

Reviewer's Responses to Questions

Comments to the Authors:

Reviewer #1: Huynh et al conducted ATAC-seq across several tissues in 8 Drosophila strains with high quality genome assemblies to understand how chromatin accessibility at cis-regulatory regions differ across tissues and genotypes. Taking advantage of the high quality genome assemblies of these lines, they inferred that sizable numbers of ATAC-seq peaks (called by MACS) that vary between strains and tissues, are false positives due to structural variants absent in the reference genome. After accounting for these issues (typically due to mapping errors) they identified a high-confidence set of peaks that vary between tissues and genotypes.

Overall, I believe this study demonstrates a very important issue regarding the use of ATAC-seq, especially given how popular the method is as a means of assessing epigenetic and regulatory changes. This is particularly problematic when comparing between genotypes, but perhaps less so when comparing between tissues. The authors provide some strategies to ameliorate the problem of false peaks, though I am not sure how widely applicable they are (see below). I commend the authors for composing a very clear, well-written manuscript, with many great examples illustrating the problem.

We agree the methods are not easily applicable in the absence of a genome assembly. This being said, in studies that compare genotypes in the absence of an assembly, the false positive rate due to the failure to account for SVs can approach 50%. So there is clear value in identifying the problem so that we can search for solutions (discussed more below).

Quantile normalization:

The authors normalized the fragment lengths between samples using a quantile normalization approach making the fragment length distribution the same across libraries. If I understood correctly, quantile normalization requires you to match the quantiles of the sample's distribution to a standard distribution. How did the authors construct the standard size distribution - i.e. how was the blue line selected to be the standard to match all the other distributions to?

It is the method described in the cited paper, widely used (especially in chip-based differential expression work). For any given sample we count the number of fragments of length L. Then over all samples calculate the mean number of fragments of length L. Finally within a sample for each length we calculate a weight (w_{iL}) such that the product of that weight and the number of fragments of length L are equal between samples. This normalizes across samples for variation for the number of euchromatically mapping reads, and further normalizes the fragment length distribution. The approach is described in the methods and git. The more general idea and advantages of quantile normalization are well-described in the microarray literature, as it is a widely accepted approach.

While the authors showed equalized fragment length distribution after quantile normalization, it is unclear to me how weighting the fragment lengths affects the intensity of ATAC-seq (or peak heights). I would like to see the variance of ATAC-peak heights between replicates before and after this normalization procedure.

It is not clear this is useful, without some sort of normalization first. For sure you want to normalize for the number of read pairs mapping to the euchromatic genome first. There is for sure variance in coverage just coming from sequencing effort (or barcoding efficiency).

Nonetheless we appreciate the general sentiment. We now include a table summarizing the “raw data” which will be helpful. We discuss such a table in the comments of Reviewer #2. We further now present the distribution of fragment lengths for all samples as a supplementary figure.

This normalization approach between replicates makes sense to me but seems less justified between tissues (and to a certain extent genotypes). It makes the assumption that all the tissues should have the same size fragmentation distribution which implies all the tissues have the same global chromatin accessibility.

The reviewer is absolutely correct here. It is equivalent to the normalization assumption in gene expression that total expression is equal between tissues (which is wrong!).

But here is the problem. Not correcting is even worse! (it took several years but eventually the gene expression folks realized this, it is now just built into most of the software used in that field, practitioners often don’t even know it is being done!). If you just look at the variation in fragment length distribution between PURE replicates (=same tissue, same genotype), now added as Supplementary Figure 8, there is considerable variation.

You see this in ATACseq datasets if you bother to carry out pure replicates – as we have here. There is a simple explanation for this variation. Small differences in how aggressive you disrupt cells, or the ratio of good nuclei to TN5, results in variation. You can do experiments with a dounce varying the

number of strokes (or vary the TN5) and show this pretty easily. The solution in the literature is to just to pretend pure technical error does not exist, and not to carry out biological replication. But that doesn't make the problem go away, it only ignores it. So we have borrowed mature methods from gene expression analysis and explicitly normalize to remove {some of} the variation associated with technical replicates.

We also think normalizing the distribution of fragment lengths makes intuitive sense. Imagine two genotypes, do you really believe the genomewide distribution of fragment lengths varies (by more than a little)? It is difficult to not believe that if you carried out 100 technical replicates per genotype, the distribution of fragment lengths would converge, and differences you see from the 3 replicates of this study is not just sampling variation. Even between tissues you are averaging over tens of thousands of peaks, again it is difficult to believe that the distribution of fragment lengths genomewide is not converging to some great extent. Albeit if you had a transcriptionally extremely active tissue versus a largely silent one perhaps there are subtle differences, but the magnitude of that effect is likely subtle compared to the sampling variation we know to be large.

Accounting for structural variants:

Because the authors have high quality genomes for the strains, they were able to identify variable peak heights overlapping SVs. First, can the author provide some examples that are not due to TEs?

There are a huge number of SVs, but TEs are the most common type of *event*. The Chakraborty et al (2019) de novo assembly paper discovered 7347 TE insertions, 1178 duplication CNVs, 4347 indels, and 62 inversions in the 94.5 Mb of euchromatin in the DSPR founders. So ~60% of events are TEs, another ~30% INDELs, and ~10% everything else.

We provided an example for an INDEL below. We have not altered the manuscript though, as we feel the examples we give are representative. Further, there are so many examples, as quantified in the manuscript, that we can't show them all or the manuscript bogs down (and we greatly edited the number of examples before submitting). Below we show a ~2kb deletion in

the A4 strain in the intron of the gene *Abl*. An ATACseq peak is contained entirely within the deletion. So based purely on short reads alignment (the 2kb INDEL is invisible to short reads) you would be led to conclude the chromatin is closed in this genotype, when really the region is just missing! Our method censors this peak.

This highlights both a problem with our methods and indeed the larger field. These structural variants that are invisible to short reads create situations where regions of the genome are simply “undefined” in some samples.

#####################

Not saying TEs are not important - they absolutely are - I am just curious to see whether the same effects hold for other SVs like smaller indels or inversion.

For sure they do!

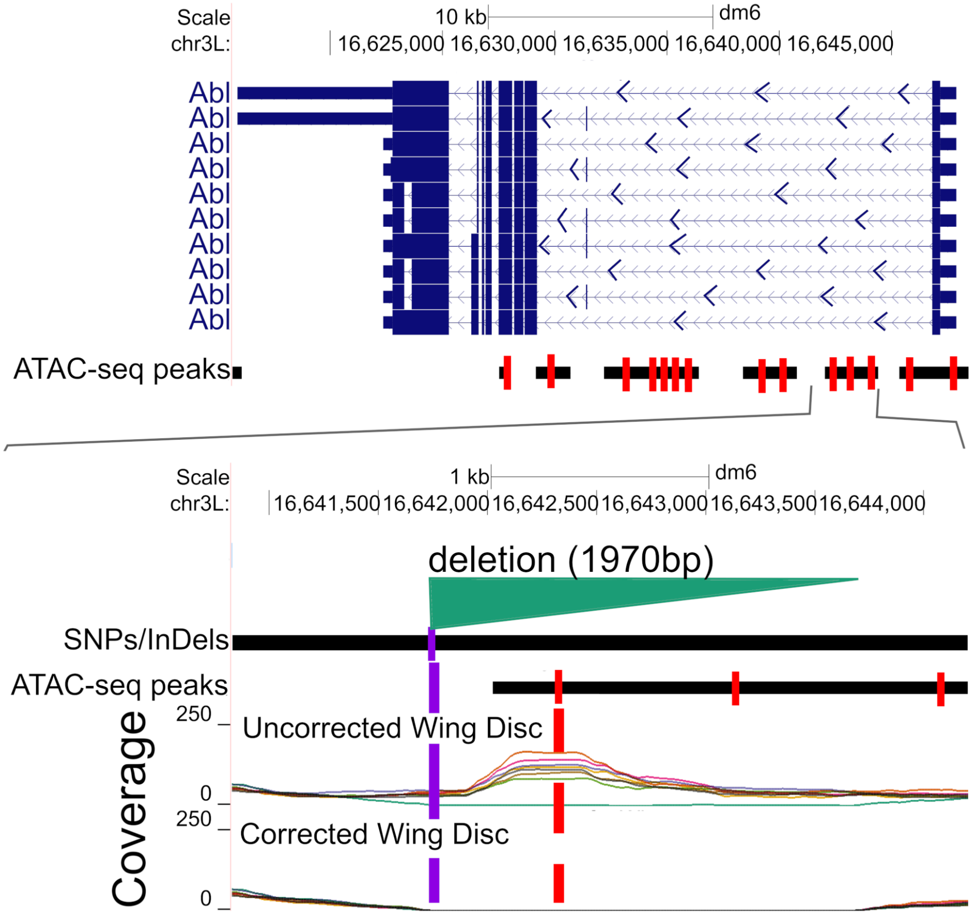

There are other aspects of structural variation that this paper only touches on. For example small INDELs (<200bp) are not scored as SVs in the Chakraborty paper (SVs below a certain size prone to false positives), but those INDELs are also missed by "SNP callers" such as GATK. Oddly, you see these events in the Santa Cruz browser if you manually look at regions with the SNAKE tracks for each genome turned on (and we provide these tracks for readers interested in a specific region). So the information is there but current tools cannot fully exploit it.

On the topic of inversions in particular. They will result in improperly mapped read pairs and a commonly employed -q 30 quality filter will remove those read pairs. So the same mismapping problem occurs. Luckily micro-inversions are not all that common in flies. But our method at least corrects for false positives associated with inversions.

On this note, I would also like to see a breakdown of the fraction of false positives (ie. those removed after SV-correction) due to different types of SVs.

This is a good idea. We have now quantified this graphically and have added this information to the supplemental materials (Supp Figure 11). We have also added a paragraph to the main text. Again, we think people just don't appreciate how common structural variants can be. In many cases there are multiple SVs within 800bp of an ATACseq peak (so the categories are NOT mutually exclusive).

We are hesitant to compare these distributions to any sort of expected proportions. If you look (for example) at ATACseq peaks totally within an SV, deletions relative to the reference dominate, while TEs are rare. This is because of the way mapping to a reference works - an ATACseq peak can only be in a TE private to the reference genome (iso1), while a deletion in any strain other than the reference will be an event (this is not easy to understand right away). On the other hand, if we look at ATACseq peaks not within an SV, but only nearby, then TEs are the most common source of false positives (consistent with their being common in the genome). So the expected distribution of false positives in the figure below are not just a function of how often each type of SV occurs in the genome, but also their site frequency spectrum, their state relative to the reference, and the way short read aligners work.

################

The need of high quality references tempers the wide-applicability of their accounting method - though I do not think it detracts from the quality of the paper. One way to also account for this would be to just use the DNA-seq from the strain as an input control and use the fold difference between ATAC-seq and DNA-input. This provides an enrichment value and is akin to ChIP-seq.

This may work. Although it is important to note that the DNAseq would need to be strain by strain. Furthermore, DNAseq coverage would have to be high enough per strain that your correction doesn't just introduce (poisson coverage) sampling error. This approach is worth exploring. But we do not feel

as part of this paper, which already covers too much ground. We hope the paper motivates people to see the extent of the problem and work towards more general solutions (than de novo assemblies of each strain). This being said, our gold standard de novo assemblies and raw data would allow any proposed method to be evaluated more fully.

And since the same mapping issues (due to TEs) or uneven mapping (due to duplications or indels) is expected to be in the input, you would expect some (but perhaps not all) of the issues mentioned in the manuscript to be mitigated without the need of a high quality reference or full accounting of all the SVs. This was previously done to look at reduced chromatin accessibility of TEs when TEs already have high coverage due to copy number (Wei, Chan, Bachtrog 2021).

Although the cited paper seems to be examining a single strain and that strain is the reference or a closely related strain. We surmise this as annotated TEs seem to be present in both the reference and the strain being examined (this would not generally be the case in *D. melanogaster* as TEs tend to be rare in allele frequency … at least those located in euchromatic regions of the genome). At any rate, this is not a great test of the proposed approach for looking between strains (where the TE is segregating and not generally annotated), or at least it is a pretty big intellectual leap. We are not saying the approach is not worth pursuing, only that considerable work would be required to develop and evaluate it the approach. We further guess that one might want ~100X of DNAseq coverage per strain characterized using ATCseq. But this is only our intuition, and the proposed approach merits fuller attention elsewhere.

Identifying causal SNPs:

It's unclear to me how the SNPs in Figure 8 can be contributing to 100% of the ATAC variation. For example in the 8A Brain sample, there is one SNP but many different heights of the peak (also the SNP does not appear centered on the peak in the schematic, despite what the text describes in Line 437). Explaining 100% of the variation means that individuals in group1 have all the same SNP variant and the exact same quantitative phenotype (ie identical ATAC distribution in this case) and individuals in group2 all have the same

SNP variant different from group 1 and have all have the same phenotype that is different from group 1.

That is perhaps one's intuition. But when you run a mixed model in R and estimate variance components, you can get results like this. It depends on model convergence properties. And it is really a ratio of variance between relative to between plus within, so depending on the magnitude of within that can drive a result.

Based on the examples, I have no doubt that you are getting at causative variants (at least causative in the association-studies sense), but I am just not sure how you are estimating the effect sizes to get to >80% in some of these instances, as there are clearly more variation in the ATAC-seq distribution that can be explained by one SNP which i presume is binary.

We do not necessarily think we are getting causative variants (see below). Only variants that are candidates to be causative (or perhaps a list of variants enriched to be causative). We tried to be clear about this. If you imagine a case where one founder is different in peak heights from the seven, then any SNP private to that founder is fantastic at explaining variation. So there is a problem with too few founders to draw robust conclusions (that we point out). This being said many feel that ATACseq peaks heights are likely controlled in *cis*, so these make for interesting candidates, as they segregate with the peak height and are physically very close to that peak. In Drosophila one might not feel so good about a variant 100kb away from a peak being able to act in cis (although there are for sure exceptions), there are plenty of examples of long range cis enhancers in mammals.

Identifying these variants could be viewed as an aggressive step (and we tried to point out the caveats). One of the reviewers did not care for this analysis. On the other hand it seems sloppy to just pretend like they don't exist. We were trying to strike a balance.

All we are doing is fitting random effects models to the data. Where we compare the peak height variation between REF and ALT alleles relative to the variation among strains (or genotypes) within REF or ALT. The ratios are variance components as estimated by the standard mixed effects model

software in R. Such variance components are not without problems, but they are a simple description of the data.

We have added some text in an attempt to clarify.

Some other minor comments and issues for clarification:

When the authors say “Genotype” in their ANOVA tests, do they mean the strain? Or do they actually mean the genotype at/around the peaks?

We use Genotype consistently throughout the paper to refer to genome-wide genotype which is equivalent to an isogenic strain. We use “marker” to refer to a locus specific genotype. That is the state of a specific SNP or SV being REF or ALT. Several genotypes could be identical in marker state (and hence genotype is nested within marker in a statistical model).

We now clarify the meaning of genotype the first time we use the term genotype in the introduction and similarly define what we mean by marker in the material and methods (and when we first fit random models in the results)

Line 344, The authors mentioned there’s no evidence for hotspots for the variable ATAC peaks, but I would like to see a breakdown of the type of gene features they reside in (near TSS, CRE, exons, introns, 5’ and 3’ UTRs etc) - ideally separately for the genotype, tissue, and GxT categories.

We include considerable information in the supplementary material. We only count peaks separately by tissue. It would be pretty busy to do this by tissue, genotype, and GxT. We also suspect GxT differences might not be meaningful.

This being said we have attempted to add a Supplementary Figure 6, that looks at peak sharing by tissue-type and feature type. So you could in theory look at these figures and get a feeling for if peaks near TSS are more likely to be shared, than those in introns. But, that being said, this is a pretty fine slicing and dicing of the data, so any observations are perhaps more hypothesis generating than confirming (I suspect we could be accussed of p-value hacking if we attempted to draw robust conclusions from these data).

But we think the reviewer's intuition is sort of bang on, there appears to be some meat on the bone here.

Line 436, Do you mean Figure 8A?

Yes

Line 720: is the 5 in C+5 used as a pseudocount? Why was 5 chosen?

It is a little arbitrary, any constant would be, but you have to add something to deal with counts of zero. Adding a constant bigger than one has the effect of making zeros less extreme in log space. That is:

```
round(log(c(1,5,1+20,5+20)),2) -> 0.00 1.61 3.04 3.22
```

So the difference between 0 and 20 is 3 if you add the constant 1 versus 1.5 if you add the constant 5. The particular constant may not matter a great deal, and peaks with zero height tend to get censored. The case where zero coverage peaks matter is a peak that is strong only in one tissue, and completely absent in another. We have added some text.

Line 745: is the "marker" variable the SNP genotype? Also what does the “1|” mean for the model?

This is standard nomenclature for mixed models in R (and I suspect *Plos Genetics* if it has such rules). In a mixed effect model the “|” or pipe is read as the factors to the right of the pipe are random effects. All the terms are to the right of the pipe in our model, making this entirely a random effects model. To estimate variance components effects have to be random (not fixed). Mixed model folks would “get” this.

The nomenclature follows this statement: “random effects model in R::lme4”. A reader understands mixed effects models or they don't. For those that do this nomenclature tells them all they need to know. For those that need to learn mixed effects models, the R::lme4 is the key package used by everyone (that hasn't moved to Julia with the author of the lme package). That package points to key books and resources.

For Figure 8, it would be great if the authors can incorporate the genotypes of the strains into the tracks. Currently there is no way of telling which Strain/SNP variant is associated with which ATAC-seq profile.

This is sort of a limitation of Santa Cruz genome browser (an otherwise amazing tool). We can and do display a SNP track, this is the way one represents a VCF in the browser, but no-one at SCGB has really championed this track, and the views are sort of dreadful. LIKE ME, THE REVIEWER WANTS PER INDIVIDUAL HAPLOTYPES!! SCGB just doesn't support this. If you turn on our SNAKE tracks representing genome assemblies (they are there in the browser just off by default), you can see all SNPs and SVs by genotype. ADL has written to the SGGB user group many times requesting an ability to color SNAKE tracks by genotype … they are not doing it yet … but how cool would this be given that we color ATACseq tracks by genotype. It starts to get busy if you have a BED for each genotype, although some sort of "vertically compressable" BED would get it done. It is very difficult to display this information in the confines of the SCGB. You would think some population geneticist surfer programmer type would relish a sabbatical in Santa Cruz!

###########

Reviewer #2: This is a very timely paper that asks whether there is genetic variation in chromatin accessibility. The question is addressed using a classical design, i.e. different genotypes of inbred strains are used to ATAC-Seq different tissues. The resulting data has 8 x 4 possible combinations of genotypes and tissues, and by replicating these treatment combinations, one can test for significance of genotype, tissue, and genotype by tissue interaction. This information is lacking in the literature in Drosophila because often only a single background/tissue is tested. I find the paper easy to read and the data set will be useful for the community. However, I do have several major concerns regarding the analyses and interpretations.

1. QC metrics should be reported for the ATAC-Seq bioinformatics, per sample/tissue/genotype, including at least, number of reads sequenced/mapped, uniquely mapped reads, number (percent) of reads within identified peaks, size (mean and sd) of peaks, etc. These pieces of information are important to understand the quality of the ATAC-Seq data and

put some of the numbers in context. For example, the numbers of peaks identified across tissues may be a function of sequencing depth.

This is an excellent idea. We have now added (a rather lengthy) table to the Supplementary material (Supp Table 4). We have further added a supplementary figure 8. The table gives metrics for each of the 96 samples of the study (total # reads, total mapped to euchromatin (before and after SV correction, pass QC filters, etc.). The figure depicts the distribution of fragment sizes for each sample, showing that even within a tissue & genotype there can be considerable variation.

2. Figure 2: In the caption, better to call the all tissues set a union rather than consensus set. For depiction of the two peaks downstream of the 3'UTR of hairy in ovaries. Based on the text, the two peaks are separate with overlapping boundaries (black bars). Perhaps it's better to separate the black horizontal bars to indicate these are two different peaks? Is the y axis really fold enrichment? This seems like a coverage plot to me, where each base gets a sequencing depth.

There are 3 points here.

First we believe consensus is a better term than union after some discussion. We consider a peak a single basepair in the genome, and MACS2 run separately on different tissues doesn't result in peaks at the exact same basepair. So we have to sort of merge peaks extremely close to one another to derive a consensus location (that we then hold constant).

Second, the black bars in the figure are those obtained from MACS2 (this is stated in the methods). So depicting them otherwise would mis-represent the calls from MACS2. The point here is that in the literature you can find examples where peaks are merged if their "boundaries" overlap. But if you have a multi-tissue dataset and are looking at non-TSS peaks where enrichment can be modest, MACS2 will often merge peaks in some tissues that are separate in others. As a result we had to develop a heuristic that didn't under- or over-merge peaks (using boundary overlaps is not good that is easy to show). Figure 2 is attempting to show the properties of our heuristic

are perhaps desirable for a set of modest but believable peaks (not the case of a screaming hot peak that is a TSS where any method works!).

The Y-axis is fold enrichment as defined by MACS2. In later plots we have coverage which is considerably higher.

3. There are other normalization methods for ATAC-Seq to deal with sample-to-sample variation in sequencing (e.g. TMM). The proposed method seems reasonable, but some additional evaluations are needed to help readers understand its performance and properties. For example, showing that the distribution of fragment size is identical after the normalization procedure (Figure 3) isn't enough to show that it works. Were PCR duplicates removed prior to the normalization? Could PCR duplicates lead to variation in the distribution of fragment sizes?

We did not look at these other methods carefully, we do not believe that they quantile normalize. Quantile normalization is WIDELY accepted in the statistical community, and was the default normalization technique for array-based expression normalization (it took years to get to that point).

We did remove PCR duplicates…as stated in the M&M around line 580: “Following this, duplicate reads are removed using picard 2.18.27 [95,96] via MarkDuplicates and REMOVE_DUPLICATES=TRUE.”

I have one concern on the assumption behind the quantile normalization method. Say under the same peak near a gene, there are 2 150-bp fragments in Sample A, 20 150-bp fragments in Sample B but 38 300-bp fragments in Sample A and 20 in Sample B. This hypothetical data would lead to equal raw coverage for this peak in both samples, but would have drastically different coverage if they are quantile normalized. Can the authors provide some intuitive explanations why in such situations identical distribution of fragment sizes is the desired outcome?

We are confused about the example case, we are not sure the reviewer totally understands the method. Quantile normalization would not necessarily make these samples equal. The fragment distributions are normalized **genome-wide** (which is why we can use single basepair length bins), not per

locus or region (or all samples would be equal!!). This normalizes method normalizes counts by length genomewide and also controls total counts per sample using weights. Figure 3 makes this most clear where the "green" line shows how the normalization makes the genome-wide coverage the same across samples. Note the Y-axis in the figure, these are counts per bin, over the entire genome.

In the example above the two 150bp fragments would each have a weight, if the particular sample was highly enriched for 150bp fragments genome-wide relative to other samples those weights would slightly downweight the observation of 2 reads, if the 150bp reads were depauparate genome-wide in this particular sample (say it was biased to nucleosome free reads) they would be slightly upweighted. The quantile normalization would have no impact (beyound correcting for total number of reads!) if the fragment size distribution genome-wide were identical for the two samples. The assumption "forced" onto the data is that all samples have equal fragment size distributions genome-wide.

The main impact of the normalization is to equally weight the distribution of nucleosome free versus mono-nucleosome, di-, tri- etc. We believe (and as discussed above it is easy to show) that much of those differences arise from sample handling artifacts. If you grind a tissue a little bit harder you shift the distribution towards naked DNA, but clearly you have to grind enough to get good cell lysis. By NOT normalizing you are equating very short with very long fragments, but fragment length means something when it comes to ATACseq data.

4. The approach to account for SVs seems to be simply blacklisting SVs. If reference quality assemblies are available, would it be possible to map reads to their own references? Then do whole genome alignments to lift the coordinates over to a single reference? In other words, you still call peaks etc. on the main reference, but for quantitative assessment of peak coverages, map reads to their own genomes and lift coordinates back to the main reference. This will not only solve the SV problem but will presumably also improve mapping quality. Note that using this approach, SNPs will also be accounted for.

Of course this is the correct way to do it! We directly address this beginning around line 512 (A potential solution would be to align reads to a genome private to each strain…). The problem is that it is not easy. Lift-overs are a great concept … that does not extend well to SVs.

One idea is to align each sample to its own private reference genome (we have this luxury). This is easy. But now to do anything you have to convert locations back to some universal coordinate system (this is where the lift-overs come in). This is easy and has been done for years for SNPs in well isolated locations … but it is more problematic for SVs. Think of a tandem dup for example, there isn't even a 1:1 mapping between genomes. It may work for a non-reference TE insertion if you are prepared to consider decimal genome locations (a TE inserted between bases 10,000,001 and 10,000,002 on chromosome 2L could have coordinates 10000001.1, 10000001.2, …, 10000001.n), but now you are completely off the rails if you want to use generic tools. Lift-overs work well for regions not near polymorphic SVs, but then you may as well mask. In a sense that is what you would be doing if you decide that there are these regions where the lift-overs are unreliable – and those regions are close to polymorphic SVs.

This problem is the impetus behind the "pangenome" methods that are being published more and more. The most sophisticated pangenome method is being developed by the SCGB people (and more specifically Glenn Hickey … who is the guy behind Progressive Cactus). We are collaborating with this group attempting to build a fly pangenome. There is promise here. But the graph cannot deal with singleton events genomewide (as the graph gets really really "tangled"). So current state of the art pangenome approaches simply censor singleton SVs (this is in the fine print of the papers, most people are not reading these sections). But here is the deal, most SVs are singletons, especially in flies where we have a better handle on the SFS than in humans. And unconditionally deleterious SVs are also far more likely to be rare. At any rate, we are back to censoring, to deal with events only present in a single founder. (I think there is promise in building a pangenome only for the set of genotypes present in a study, and then properly exploiting that, but this is a research problem in its own right).

In the end pangenome methods work well for SNPs in well behaved (non-repeat, not near SVs) regions. They are also working increasingly well for intermediate frequency complex events (this is a bit of a break-through, the pan-genome software implementations would just crash 5 years ago if given our genome assemblies to think about). But they are not quite there more generally yet. We are actively pursuing this. It is not simple.

5. I do not think any of the analyses can claim causality for SNPs. In particular, variance partitioning using mixed models cannot determine causality.

We agree 100%. We are careful to state this in the Methods section that introduces this model fitting. And we further re-iterate this in the Discussion. We clearly state what the model is doing.

"We identify several thousand such SNPs that explain more than 80% of the variation due to genotype or a genotype by tissue interaction for coverage, a collection likely enriched for causative polymorphisms, despite some potential for over-fitting."

This being said, we do not want to throw the baby out with the bath-water either. Many people believe that the factors controlling chromatin modification are likely encoded in *cis*, and physically close to the peaks they control. This is an entire cottage industry in human genetics where GWAS hits near chromatin mods are often considered as candidate causative variants (based on the "weight of the evidence"). This is essentially the same exercise.

The true test is obviously experimentally editing bases, which would be beyond the scope of the current study. That said, I do believe that cis variants can cause polymorphism in chromatin structures, but it's a hypothesis that is best tested using other designs.

Clearly, the real test is a replacement!! As the reviewer points out this is beyond the scope of the paper. It is not being routinely done in flies right now. It is close to impossible in mice still. And clearly is not even considered in humans (beyond cell culture). Oddly there are few examples even in yeast. But for sure this is where the field has to go.

The replacements are hard to do in controlled backgrounds…we are working on it…as are others. To be brutally honest a site replacement for a single SNP in an otherwise isogenic background is an eLife paper on its own right now. (the reviewer agrees).

Another caveat I want to point out is, if SNPs are not accounted for during mapping, there is a serious confounding issue because proximal SNPs may affect mapping of reads themselves. In other words, the causality may not be biological but mapping related artifact.

It turns out that with the longer PE reads we are working with these days and bwa-mem, reads failing to map due to SNPs is much rarer than it was in the olden days (with shorter reads, often non PE reads, and crappier aligners). I am not sure this is widely appreciated. We have done experiments (not part of this paper) where we map reads to the strain specific genome, and then compare the number of mapped reads to those mapping to the reference using bwa-mem. For well behaved single copy regions not near SV regions (i.e., what is considered in this paper) bwa-mem is mapping close to 100% of the reads. Where you are getting mapping failure is near SVs (a point of this paper), regions where you have dense clusters of SNPs (say 10 SNPs in a read), or these smaller INDELs (that are too small to pick up as SVs and too large to be called correctly with GATK so are hidden to short read callers).

I don't think this section is necessary, it actually weakens the paper.

We are on the fence. We debated leaving it in or taking it out. We decided that people would want to see this. But we agree that we have not proven they are necessarily causative – which we clearly state. I bet we could pull up papers in Plos Genetics that do similar things to this in humans (I sure can in Nature Genetics and eLife … part of the new GWAS paper "formula"), that is claim SNPs physically close to a chromatin mod that segregate with it are causative (without nearly our level of caution). Just look at the highly cited omnigenic hypothesis paper. So there is a question of standards.

I am happy to defer to the Associate and Section editors. But we feel we are OK given we state the limitations, and are careful to refer to these as “candidate causative”.

##################################

Reviewer #3: In the work by Huynh et al., the authors sought to study the genetic variation in the chromatin state in Drosophila melanogaster. To achieve this goal, the authors sequenced and analyzed a number of ATAC-seq data from eight strains and four tissues. They performed a rigorous analysis of the project and had a number of interesting discoveries. The paper is well written; the dataset will be of great interest to many readers. The authors could have dug into some of the interesting observations further, but it is their decision. I only have a few minor comments.

1. One of the surprising findings is that 65.6% of the peaks are private to a single tissue. This is very interesting, but brain data largely affects the result. I’d recommend the authors also report the tissue-specific peaks by tissues. For example, eye and wing discs have a much lower percentage of tissue-specific peaks.

This is an excellent point. We have added this information to the supplement (Supp Figure 6).

2. The authors found that most peaks have fold enrichment of less than 5. What does that mean? Is it because most peaks have residual open chromatin regions nearby, or because most of the peaks only exist in a small number of cell types within a tissue (e.g., cell type-specific peaks with tissue-level background noise)? I understand the authors’ data may not give a definitive answer. Nevertheless, it would be interesting to check it.

Our guess is that it is sort of a big dataset, so MACS2 can call more peaks confidently at lower enrichment scores. That is the bigger the dataset the smaller the enrichment score that MACS2 is confident above background. Our guess is perhaps the same as the reviewer in that a peak only seen in one tissue may in-fact show less fold enrichment.

An odd thing about ATACseq data, that is well understood by practitioners, but perhaps less appreciated more generally, is that the absolute strongest peaks are associated with TSSs/5'UTR/TTS. So despite the idea that ATACseq is somehow uncovering the cis-regulatory regions of genes, the peak height for what is thought of as traditional cis-regulatory elements a kilobase or more upstream of the TSS often have much more subtle fold enrichments. I would argue that these are also the "peaks" that are more likely to be tissue specific (TSSs for most genes are just open). This is now explored in the supplementary figures. But we have resisted growing the paper, as any conclusion would seem speculative.

3. It is unclear how many of the peaks located in exon/UTR/intergenic regions in each tissue are shared. For example, it is likely that promoter/TSS regions of universally expressed genes are shared by other intergenic peaks are more tissue specific. Maybe I missed it somewhere in the manuscript, but it would be interesting for readers to know.

This is dealt with in the new Supplementary Figure 6 discussed above.

4. The authors provided a nice example: without considering TE-related indels, the comparisons can be screwed. Is it a rare case? In total, how many such TE insertions are found in the eight strains?

In the 14 DSPR founders there are 7347 TE insertions, 1178 duplication CNVs, 4347 indels, and 62 inversions in the 94.5 Mb of euchromatin in the DSPR founders, since the vast majority of events are private, the strains here would have roughly half these numbers. TEs are the most common SV event (~60%), INDELs the second most common (~30%).

We show in this paper that if we ignore these events and just compare peak heights among genotypes we have close to a 50% error rate, where we conclude a difference in heights among genotypes, that is really due to mismapping due to a SV. The vast majority of such events are due to TEs and INDELs, as they are relatively more common relative to the other events. We have added text to the paper. We also show an example of an ATACseq peak located totally within a polymorphic non-TE INDEL earlier in this response.

5. Line 424-428, I am not sure if I understand “a total of 22 SNPs annotated as having a high functional impact”. Why would SNPs with protein sequence or structures (missense, premature start codon, or splice variant) could fully explain 100% of the peak height? It is theoretically possible to have linked regulatory SNPs in the nearby regions, but the authors do not seem to suggest so.

We agree. It doesn’t make a great deal of sense. It is only what we observe. Sometimes SNPs annotated as having high functional impact are problems with the annotation. These are just the SNPs that pop out of the analysis we do, where we require that the SNPs be physically close to a polymorphic peak. Since many ATACseq peaks are close to TSS, perhaps it should not come as a surprise that a nearby SNP is exonic.

Of course the analysis we do only looks at SNPs extremely close to a polymorphic ATACseq peak. So there could be SNPs in complete LD a little further away that are in a perfectly good TFBS sequence. But the argument in the field is that SNPs closer to the peak are better candidates, but maybe 10kb away is totally reasonable.

We have added text in an attempt to clarify.

6. It is unclear to me, in the method, how the authors accounted for structural variants. I understand the authors have de novo reference genomes for each strain. With indels, the coordinates will be different at all locations on the genome. How the authors used de novo genomes and provide information for standard genomes?

We map to the reference genome. We then exclude ATACseq peaks within 800bp of a SV (or contained within an SV) present in any of the 8 strains. 800bp is roughly the distance over which mismapping of reads occurs. So we exclude all ATACseq peaks physically close to a polymorphic SV.
