# Supplementary material for: Genetic variation in chromatin state across multiple tissues in Drosophila melanogaster
Source: PLoS Genet. 2023 May 5;19(5):e1010439. doi: 10.1371/journal.pgen.1010439 (PMC10191298; doi:10.1371/journal.pgen.1010439)
Supplement: Attachment — Submitted filename: Response_2.pdf [file pgen.1010439.s021.pdf]

Dear Kelly:

Thank you for the opportunity to hopefully do a final round of revisions on this manuscript. The changes were minor enough that I only provide a standard double spaced version of the manuscript with separate figures and track-changes on. I feel this makes your job the easiest. Most of the changes are in the discussion (to accommodate Reviewer#1).

First, we have now placed the "tarball" we were self-hosting here: https://doi.org/10.7280/D1FM5F and documented this in the manuscript. I promised this change to the journal.

We hope the final changes detailed can accommodate Reviewer#1 as much as possible. We do not disagree with his comments at all, but are trying to strike a balance of including everything necessary and not having the manuscript blow up and be too long and impenetrable.

Reviewer #1: The authors addressed most of my concerns and questions, but I have some lingering issues that the authors should clarify.

Quantile normalization of fragment lengths:
I had asked the authors to demonstrate how their quantile normalization affects peak calling and peak intensity. The authors replied that this is not useful, and mentioned they provided supplementary table 4 with mapping stats and supplementary figure 8. The point of my comment was that the authors do not show how the quantile normalization strategy affects downstream inference of chromatin accessibility (i.e. the process of calling peaks and quantifying intensity/accessibility). Does this manipulation/normalization make the ATAC-seq signals (peaks and peak intensity) more robust and less variable? How does peak intensity (or even coverage over peaks) change using the quantile normalization procedure vs. just normalization by library size. Are these values more or less variable between replicates in this normalization scheme compared to more rudimentary methods? The question I am getting at is whether quantile normalization produces more robust quantitative and qualitative characterization of accessibility and chromatin states. As far as I can tell, the authors merely demonstrated that their quantile normalization procedure makes the fragment size distribution more robust - but the more meaningful question is whether accessibility measures are more robust.

The reviewer brings up two issues here: Peak Calling and Peak Intensity. The normalization method has absolutely no effect on peak calling. This is clear from the methods, and Supplementary Figure 2 which describes our pipeline as a graphic. Peak calling is done on data pooled by tissue using MACS2 and happens prior to any normalization.

Of course the reviewer is correct that the normalization could impact peak "intensity" or peak "heights", given a called peak. This would manifest as differences in means among replicates, tissues, or genotypes. This could lead to different p-values when one carries out an ANOVA (or any statistical test). The reviewer wishes for us to quantify the impact of normalization, but we

feel that would be a distraction, and it is unclear how to quantify at any rate. There are almost certainly going to be differences arising from the normalization. But lacking a ground truth, how do we know if un-normalized or normalized results are better? We feel instead one has to look at the fragment size distribution plots, which are an important QC step in the field (i.e. they are featured in the original ATACseq paper from the Greenleaf lab, and also the main R package people use to look at these data (ATACseqQC)). If one sees differences within pure-replicates in the fragment size distributions it is difficult to believe that those differences are biological, especially given that we can demonstrate that more aggressive processing of nuclei shifts the fragment size distribution. So once you make this observation that there appear to be quantitative differences in fragment length profiles among preparations, most statisticians would normalize the counts by legnth to control for this sample prep problem (just as it is obvious to normalize by # reads).

One could accept the normalization approach, but still feel it is important to quantify the magnitude of its effect for other users. We are concerned that this could do more harm than good. The degree to which normalization impacts inference in some other study depends on how similar the fragments distributions are between samples. We imagine there are labs/tissues that are super amenable to ATACseq preps (if you talk to people in the field cell lines work better than tissues), in that case perhaps no correction is necessary. On the other hand, a more difficult tissue (we find malphigian tissues extremely difficult to work with), and/or a less competent technician will result in larger differences in those fragment size distributions. At some point people in the field look at these distributions for each sample and discard a subset as being a "failed prep", and then use the "successful preps" without additional correction (this is implicitly acknowledging the continuum we correct for). Our proposed corrector is likely only effective in normalizing libraries that investigators generally consider "good", but different people have different standards. The result is that the degree to which normalization impacts inference is going to be highly variable between studies, so it would be somewhat reckless to assign a number (from this dataset) to that impact.

Our feeling is that the correction should be put out there. And then its impact would be better addressed in a more focused study. Its impact would be best assessed in a "meta-analysis" that looks across several published datasets, and would be especially impactful if focused on (the not so common) collection of datasets with pure replicates. Doing more here would make an already too long paper less focused.

We have added some material to the Discussion to reflect these ideas.

Regarding whether tissues should have the same fragment size distribution, I agree mostly with the authors that most tissues are probably quite similar, therefore justifying the use of fragment size distribution (quantile) normalization, at least in their case. But there are instances where this assumption is problematic - early embryo development and zygotic genome activation when the chromatin landscape undergoes major changes. The chromatin environment during this time is of particular interest to a lot of researchers, and subject to MANY ATAC-seq studies. I highly

recommend the authors caveat their approach and mention when the assumption (of similar fragment size distribution) may not be met.

We have added material addressing this point to the Discussion. The reviewer is correct, normalizing between tissues could potentially hide real biological differences.

But, to be totally honest, embryos are pretty trasncriptional complex. We guess normalization would be helpful for this tissue. Of course we generally agree with the reviewer that ANY normalization method is likely to be more robust for within tissue comparisons. Between tissue comparisons are potentially prone to artifacts from a normalization method. This being said, differences between tissues are so much more dramatic than within, that you hardly need statistics. And we feel our normalization method was helpful as we were interested in comparing genotypes within tissue. Furthermore we had pure replicates, which are rare in the field. And pure replicated clearly illustrate that much of the variation we see in fragment sizes is due to sample prep.

Example of SVs causing false peaks.
Upon my request of including a SV not related to TEs, the authors provide one example of a 1970bp deletion causing a false peak. I am a bit confused by this example since as the authors mentioned this leads to the erroneous inference of closed chromatin. But in the ATAC-seq tracks they show, in the uncorrected track, the deletion is associated with an increased ATAC-seq signal (and a downstream peak). Also, I would highly recommend that the authors include at least one of such indel-associated issues in the supp, instead of only supplying for reviewer eyes.

The point of the figure is that the “green” genotype harbors a 1970bp deletion. The figure (reproduced below) was drawn misleadingly, as the green triangle should have been inverted (we have now fixed this). This ~2kb deletion would generally be invisible to short read datasets/aligners, but knocks out two ATACseq peaks. So what an investigator would see is the green strain having closed chromatin relative to the other 7 open chromatin strains, with the much smaller differences between non-deletion strains likely not significant. The closed chromatin inference is of course totally wrong, the problem is the hunk of the genome with the peak is just missing.

This being said, the reviewer is getting at a problem. We are saying the inference of closed chromatin is incorrect, since the hunk of the genome harboring the peak is essentially missing. But one could argue if the peak is missing then it is also closed. This misses the bigger picture, short reads are giving a misleading picture of what is going on in this region, so we mask SV regions.

We have now included a corrected figure for this region as a supplement at the reviewer’s request.

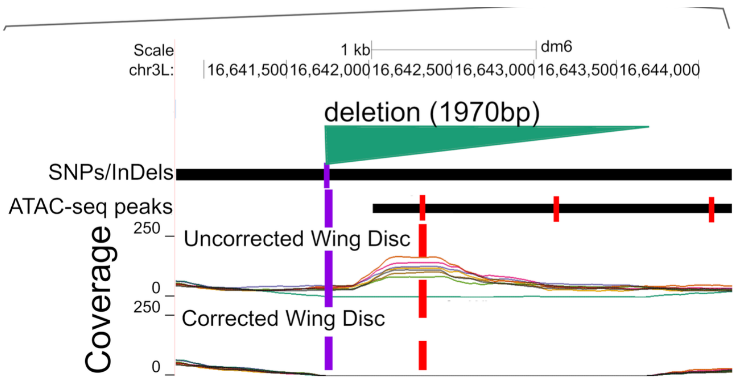

Scale
chr3L:
1 kb
dm6
16,641,500
16,642,000
16,642,500
16,643,000
16,643,500
16,644,000
deletion (1970bp)
SNPs/InDels
ATAC-seq peaks
Coverage
250
0
Uncorrected Wing Disc
250
0
Corrected Wing Disc
